# The global regulator LaeA controls expression of genes involved in diverse primary and secondary metabolites biosynthesis and their secretion in fungi

**DOI:** 10.3389/fmicb.2026.1764309

**Published:** 2026-03-09

**Authors:** Juan Francisco Martín, Katarina Kosalková, Paloma Liras

**Affiliations:** 1 Departamento de Biología Molecular, Área de Microbiología, Universidad de León, León, Spain; 2 Área de Biotecnología, Cesefor Parque Científico de León, León, Spain

**Keywords:** fungi, LaeA, metabolites secretion, pathogenicity, secondary metabolites, siderophores, VeA, velvet complex

## Abstract

LaeA is a global transcriptional regulator that differentially modulates expression of an impressive variety of secondary metabolite gene clusters in fungi, acting as a positive regulator fo some while repressing others. This regulator controls the production of fungal cellulolytic/ligninolytic enzymes and citric acid secretion in Ascomycetes. Also, it regulates the biosynthesis of siderophores and the antitumor agent ganoderic acid in Basidiomycetes. This regulator contains an S-adenosylmethionine-binding motif, suggesting a function as a methyltransferase, although its specific methylation substrate has not been identified. LaeA is a core component of the velvet complex, a five-membered proteins assembly that regulates fungal responses to light and environmental stresses. Within this complex, LaeA interacts with VeA, VosA and other velvet components forming dimers and trimers and the complexes bind to an eleven-nucleotide consensus sequence in the promoter of target genes. This article reviews current knowledge of LaeA and VeA mediated regulation, discusses outstanding challenges in the field, and highlights its potential in optimizing fungal metabolism, its biotechnological applications and its role in the control of fungal infections.

## 1 Introduction

### 1.1 Early findings on LaeA regulation of secondary metabolism in fungi

**Figure 1:**
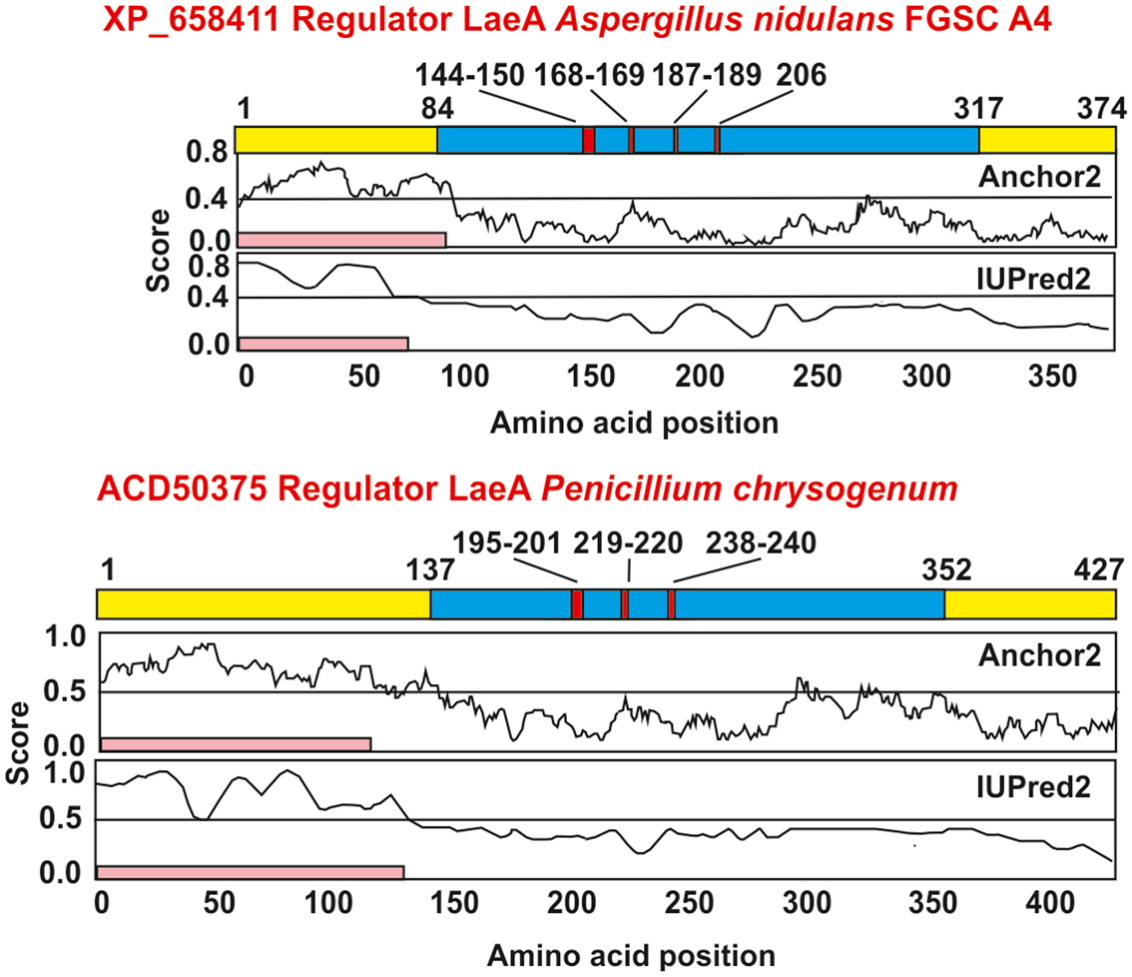
Sequence based analysis of protein domains, motifs, and disordered regions of LaeA. LaeA sequences from *Aspergillus nidulans* (upper scheme) and *Penicillium chrysogenum* (lower scheme) were obtained from the protein database of NCBI (https://www.ncbi.nlm.nih.gov/). Domains and motifs in LaeA proteins were obtained from the InterPro database (https://www.ebi.ac.uk/interpro/) (Blum et al., 2025). LaeA proteins are shown in yellow, whereas the domains are colored in blue and motifs are colored in red. Below a pink band indicates the disordered N-terminal region of LaeA according to the IUPred2 and the ANCHOR2 servers (https://iupred2a.elte.hu/) (Erdős and Dosztányi, 2020); Mészáros et al., 2018).

The *laeA* gene, so named for “lack of *aflR* expression” was first reported by Bok and Keller (2004) in *Aspergillus nidulans* and *Aspergillus fumigatus*. Complementation with *laeA* of a *A. nidulans* mutant defective in sterigmatocystin biosynthesis showed that LaeA has a positive effect on the biosynthesis of different secondary metabolites (SM), as sterigmatocystin and penicillin in *A. nidulans,* and gliotoxin in *A. fumigatus.* LaeA also controls heterologous expression of lovastatin genes subcloned in *A. nidulans*. The regulatory factor LaeA in *A. nidulans* has 365 amino acids, and contains a S-adenosyl methionine (SAM) binding site, present in methyltransferases, but lacks a SET domain. The SET domain is characteristic of histone metyltransferases (Veerappan et al., 2008; Freitag, 2017; Lai et al., 2022) and its absence in LaeA suggests that this protein is not a true histone methyltransferase. A diagram of *A. nidulans* LaeA protein showing the S-adenosyl-methionine dependent methyltransferase domain and the S-adenosyl binding site motifs is shown in Figure 1; information on different fungi LaeA proteins is shown in Supplementary Table 1.

Transcriptional studies indicate that LaeA activates expression of all genes of the sterigmatocystin gene cluster but no genes in regions adjacent to the cluster (Bok and Keller, 2004). These authors showed that the LaeA protein is located in the nucleus, as occurs with many other regulatory factors, and its regulatory effect is observed at the transcriptional level of the target genes.

### 1.2 LaeA is a putative methyltransferase: which is the substrate(s) methylated by this enzyme?

The LaeA conserved S-adenosylmethionine (SAM) binding motif is present in all known LaeA homologs across diverse fungal species; this led to the proposal that LaeA functions as a histone methyltransferase. Many enzymes utilize SAM as cofactor or as substrate in a variety of regulatory and biosynthetic reactions unrelated to methyl transfer (Fontecave et al., 2004). The versatility of SAM stems from the strong electrophilic character of the S–CH₃ group, which enables methyl transfer to substrate proteins (Patananan et al., 2013).

Interestingly, the *A. nidulans* LaeA protein has been reported to undergo self-methylation at a methionine residue located near the SAM-binding site, forming an S-methyl-methionine modification. This self-methylation may render LaeA chemically reactive, as the S-methyl-methionine residue could potentially act as an enhanced methyl donor under certain conditions (Patananan et al., 2013). Nevertheless, experimental attempts to identify substrate proteins methylated by LaeA—using radiolabeled H^3^-methyl-SAM—failed to detect any other protein methylated by LaeA in *A. nidulans* (Patananan et al., 2013).

Bioinformatic analyses have predicted that LaeA localizes to the nucleus, a finding corroborated by experiments using GFP-fused LaeA fluorescence microscopy (Bok and Keller, 2004; Bayram et al., 2008). This nuclear localization is consistent with its alternative role as a transcriptional regulator. Moreover, expression of *laeA* itself is regulated by other transcriptional factors; for instance, in *A. nidulans*, the binuclear Zn(II)₂Cys₆ transcriptional factor AflR. This factor downregulates *laeA* expression and in turn LaeA activates expression of the sterigmatocystin gene cluster (Bok and Keller, 2004). This suggests a regulatory cross-talk between different transcriptional factors that collectively influence SM biosynthesis.

## 2 Transcriptional regulation by LaeA involves the velvet protein complex

There is increasing evidence in *A. nidulans* showing that the global regulator LaeA exerts its function through interactions with other components of the velvet complex. Notably LaeA, VeA and VelB, form what is known as the core velvet complex, as confirmed by mass spectrometry analyses (Bayram et al., 2008; Bayram and Braus, 2012).

Detailed studies of these interactions revealed that VeA serves as a bridge protein linking LaeA and VelB forming the trimer VeA-LaeA-VeB. Specifically, VeA interacts directly with LaeA, while its N-terminal region mediates binding to VelB.

In *A. nidulans*, both sexual development and SM biosynthesis are tightly regulated by light (Bayram et al., 2008). Under light conditions, VeA is predominantly located in the cytoplasm; however, in the dark, VeA is translocated into the nucleus, where it forms a complex with VelB and the global regulator LaeA (Stinnett et al., 2007). The nuclear localization signal (NLS) located in the N-terminal region of VeA is recognized by the nuclear import factor KapA, which mediates VeA transport into the nucleus in the absence of light (Stinnett et al., 2007). Very recently, an important contribution to understand the transport of the VeA protein of *A. nidulans* into and out of the nucleus has been reported (Strohdiek et al., 2025). VeA plays a key role in both the asexual and sexual differentiation. This protein contains nuclear localization signals (NLS1, NLS2 and NLS3) that contribute to the transport of VeA to the nucleus. VeA is located in the nucleus during vegetative growth. The permanence of VeA in the nucleus correlates with the progress of sexual differentiation but VeA also has an export signal (NES) that allows it to be secreted to the cytoplasm what favors asexual sporulation. This work shows that an accurate control of the export and import of the VeA protein is critical for the coordination of growth, sexual and asexual differentiation and the biosynthesis of SM (Strohdiek et al., 2025).

It is still unclear whether LaeA directly recognizes specific DNA sequences within the promoters of the regulated genes or if this function is instead mediated by the trimeric velvet complex VeA-LaeA-VelB (see Conclusion and Future Outlook, section 14). The VosA, VeA, VelB, and VelC components of the velvet complex—but not LaeA—contain a 150-amino-acid DNA-binding region known as the velvet domain. Binding of the velvet complex to a two 11-nucleotide consensus motifs within the promoter of asexual development regulatory genes such as *brlA*, *wetA*, and *vosA*, has been suggested to regulate their expression (Ahmed et al., 2013). The velvet domain is essential for DNA recognition as shows the crystal structures of VosA and the VosA–VelB heterodimer, and by site-directed mutagenesis of the encoding DNA (Ahmed et al., 2013). Summing up, these authors proposed that transcriptional regulation in fungi may be mediated by different combinations of velvet components homo- and heterodimers, each activating distinct sets of target genes.

### 2.1 LaeA and VosA act as mediators of the mitogen-activated protein kinase MpkB that phosphorylates VeA

**Figure 2:**
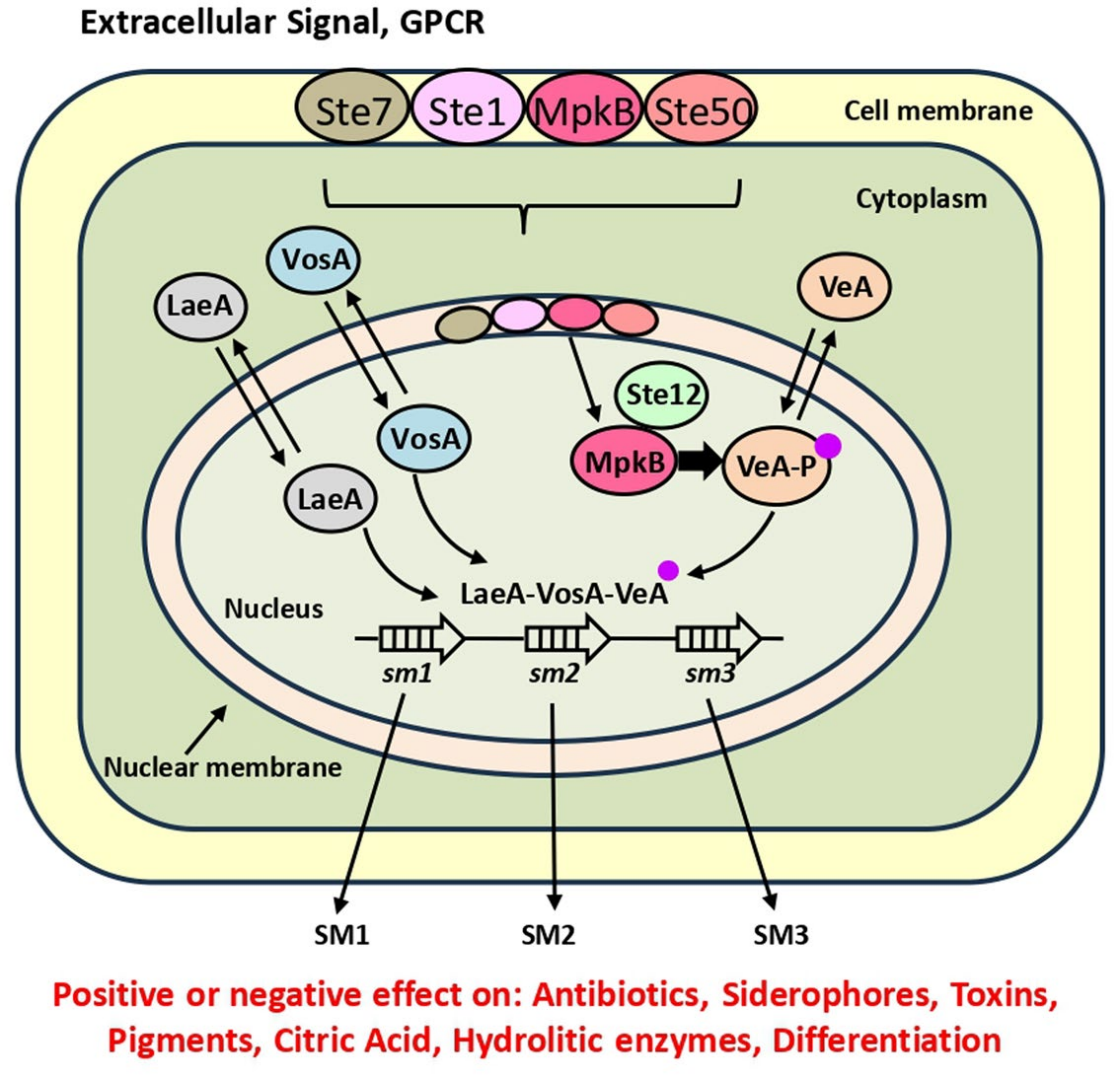
Comprehensive view of the mechanism that controls expression of SM gene clusters by LaeA and its interaction with the MAP kinase cascade. The MAP protein kinases shown first at the cell membrane level, after receiving a GPCR signal move to the cytoplasm and then to the nuclear membrane (Kang et al., 2013; Bayram et al., 2012; Frawley et al., 2020). The MpkB kinase enters into the nucleus, interacts with the transcription factor Ste12, and phosphorylates VeA (the phosphate group is shown as a small pink dot). Phosphorylated VeA interacts with LaeA and other velvet proteins. The dimeric/trimeric complex induces or represses SM1, SM2, SM3 gene clusters indicated by open white arrows.

Filamentous fungi are able to regulate their metabolism by several signaling pathways in response to sensing very diverse extracellular environmental or nutritional signals, including cell to cell communications (Fischer and Glass, 2019; Figure 2). One of the best-known signaling pathways is the MAP kinases-mediated pathway, which functions through a cascade of phosphorylated proteins. This cascade, conserved in all eukaryotic organisms, includes three mitogen activated protein kinases named MAPkkk, MAPkk and MAPk that activate each other by phosphorylation following sensing of signals at a membrane receptor. The extracellular cues are sensed by specific GPCRs (G protein coupled receptor) (García-Rico et al., 2008; Martín et al., 2019). The third member of the cascade, MAPk, after being phosphorylated enters into the nucleus where interacts with transcriptional factors thus regulating multiple cellular responses. The best-known protein kinase in yeasts is the so-called pheromone module FUS3 that in response to extracellular pheromones is phosphorylated at the membrane level and the phosphorylated form enters in the nucleus interacting with the Ste12 transcriptional factor (van Drogen et al., 2001; Bardwell, 2005). As result of this interaction the cell regulates sexual mating (Bardwell, 2005; Wong Sak Hoi and Dumas, 2010).

In the filamentous fungi *A. nidulans* the MAPkinase cascade consists of the three components Ste7, Ste11 and MpkB, the last one analogous to FUS3 (78% identity) and the adaptor protein Ste50 but it lacks the scaffold homolog to Ste5 that exist in yeasts (Bayram et al., 2012). In this fungus the MAPkinase complex is located at the cell membrane where it is activated by a sensor GPCR. The phosphorylation is transmitted by a phosphorelay to MpkB. This kinase enters the nucleus and interacts with the transcriptional factor Ste12 and phosphorylates the velvet component VeA (Figure 2) triggering fungal differentiation and SM biosynthesis. Importantly, MpkB is necessary for normal expression of the *laeA* regulatory gene. This finding suggests that the effect of MpkB is at least partially mediated by the LaeA global regulator since the absence of MpkB has a similar effect to that exerted by the deletion of *laeA*. These studies were later extended showing that the MpkB kinase is also required for expression of *vosA* and of the *brlA* gene that encodes a zinc finger transcriptional factor essential for asexual development (Kang et al., 2013). These authors suggested that the role of MpkB is mainly exerted by controlling the timely expression of *brlA* in *A. nidulans.* The MpkB protein kinase controls the production of autolytic enzymes, such as quitinases and glucanases, that are involved in remodeling of the cell wall maintaining its plasticity; this is important for spore germination, hyphae growth and mycelium branching. Deletion of *mpkB* results in premature germination as it was also observed in a VosA-defective mutant (Ni and Yu, 2007). These findings stablish a connection between the MAP kinase and the velvet complex regulation. An *A. nidulans mpkB*-defective mutant has lower production of sterigmatocystin and other SMs such as penicillin and terraquinone (Atoui et al., 2008; Bayram et al., 2009). Recently in *Aspergillus flavus* has been found a similar tetrameric kinase complex, that consists of three kinases and the adaptor protein SteD (Frawley et al., 2020; Jun et al., 2020). In this fungus the tetrameric complex is assembled in the cytoplasm probably attached to the cell membrane and then the MpkB is translocated into the nucleus. Deletion of MpkB or the SteD components resulted in the loss of asexual spores and cleistothecia, reduced production of aflatoxin B and increased formation of other SM (Frawley et al., 2020). These results suggest that the tetrameric complex is conserved in these *Aspergillus* species but there is still no information about the possible conservation of the same components in taxonomically distant filamentous fungi.

Studies in a different fungus, *Fusarium graminearum,* showed that a MAP kinase, encoded by *mgv1,* regulates mating (particularly female fertility), heterokaryon formation, and production of toxins involved in plant infections (Hou et al., 2002; Paoletti et al., 2007; Schalamun et al., 2024). Different kinase cascades, such as the high osmolarity glycerol (HOG) cascade, exist in other filamentous fungi, e.g., *A. fumigatus* (Ma and Li, 2013). In summary, there is a clear effect of MpkB on the expression of the global regulatory proteins.

## 3 LaeA and the velvet complex regulation of secondary metabolites and differentiation in *Aspergillus* species

The role of LaeA and other velvet components has been also extensively studied in three other *Aspergillus* species: *Aspergillus parasiticus*, *A. flavus* and *A. fumigatus* (Calvo et al., 2004; Kale et al., 2008; Perrin et al., 2007). The first two species are taxonomically close, sharing more than 90% of their genomic content, and the biosynthesis of aflatoxins has been well characterized in both of them (Linz et al., 2014). Aflatoxin B₁, a highly potent carcinogenic compound, is produced by these fungi when they infect food crops, e.g., corn and peanuts.

### 3.1 *Aspergillus parasiticus* and *Aspergillus flavus*

The aflatoxin gene cluster of these fungi comprises 27 genes tightly packed within a 17 kb DNA region (Yu et al., 2000; Ehrlich et al., 2005). The biosynthetic pathway and regulatory mechanisms controlling aflatoxin production in *A. parasiticus* have served as a model for studying and characterizing the regulation of the biosynthesis of many other SM pathways. Regulation of aflatoxin biosynthesis is mainly controlled by a cluster-situated binuclear Zn(II)₂Cys₆ DNA-binding protein, AflR, that functions as a positive regulator of many aflatoxin genes (Price et al., 2006; Kale et al., 2008).

Initial studies on the role of the velvet proteins in *A. parasiticus* demonstrated that VeA controls the biosynthesis of several SMs, including aflatoxins, and also regulates the formation of sclerotia (Calvo et al., 2004). These findings were later confirmed and expanded showing that, in addition to controlling sclerotia formation, VeA positively regulates the production of aflatoxins, cyclopiazonic acid, and aflatrem (Duran et al., 2007).

Bioinformatic analysis of the *A. flavus* genome allowed the identification of the *laeA* gene by comparison with its homologs in *A. nidulans* (75% identity) and *A. oryzae* (100% identity). Transcriptional analyses revealed that *laeA* negatively regulates expression of the *veA* velvet partner gene, a crucial cross-talk, given that VeA plays an important role in the regulation of secondary metabolism (Kale et al., 2008). Using *A. flavus* strains containing 0, 1, or 2 copies of the *laeA* and *veA* alleles, it was found that both *laeA* and *veA* deletion mutants were unable to metabolize host lipids and exhibited poor growth on infected seeds (Amaike and Keller, 2009). Interestingly, the ratio of sclerotia to conidia in cultures depended on the copy number of *laeA,* but not on that of *veA.* In summary, the deletion of *laeA* confirm that LaeA acts as a positive global regulator of secondary metabolism, particularly for metabolites associated with sclerotia formation. Similarly, conidia production using pine seeds as substrate was reduced in the *laeA* mutant and increased in the overexpression strain.

### 3.2 Aspergillus fumigatus

Early parallel studies on the role of *LaeA* focused on its characterization in *A. fumigatus*, an important pathogen infecting both humans and immunocompromised mice (Perrin et al., 2007). The effect of LaeA on the pathogenicity of *A. fumigatus* is more complex than in *A. nidulans*, as deletion of *laeA* affects the biosynthesis of multiple pathogenicity factors. However, a mutant specifically defective in gliotoxin biosynthesis shows no major reduction in pathogenicity, suggesting that the role of LaeA extends beyond its regulation of gliotoxin production.

A comprehensive study comparing the SM profiles of an *A. fumigatus laeA* mutant and its parental strain revealed that 13 out of 22 SM gene clusters are regulated by LaeA (Perrin et al., 2007). This represents a positive regulation of approximately 20–40% of the SM biosynthetic genes, including those encoding non-ribosomal peptide synthetases (NRPS), polyketide synthases (PKS), and cytochrome P450 monooxygenases, which are known to participate in the biosynthesis of toxins, melanin, and other SM. Notably, one of the LaeA-regulated SMs is a siderophore encoded by the *nrps2* gene, whose expression is markedly reduced under iron-limiting conditions. Siderophores play a critical role in fungal metabolism since they perform iron scavenging from the environment and transport of this metal ion into the fungal cells. During fungal infection the siderophores disturb the iron homeostasis of the infected tissues. Due to the essential role of iron metabolism for fungal survival during human infection, this finding suggests that certain aspects of LaeA-mediated regulation—such as the control of siderophore biosynthesis are of great importance to *A. fumigatus* pathogenicity.

Using different strains of *A. fumigatus*, some of them virulent clinical isolates, it was observed that a disrupted *laeA* mutant showed a significantly reduced (by 80%) expression of the *glp* gene involved in the formation of gliotoxin and produced conidia with a smooth surface (Sugui et al., 2007). This absence of surface protuberances, probably due to the lack of expression of the *alb1* gene encoding a PKS, is associated with increase susceptibility to the host phagocytosis (Sugui et al., 2007). The LaeA mediated regulation of phagocytosis of *A. fumigatus* spores is associated with a modification and decrease in the hydrophobicity of the cell wall surface (Dagenais et al., 2010).

## 4 LaeA regulation of *β*-lactam antibiotics

The global regulator LaeA controls the biosynthesis of β-lactams in different fungi, including *Penicillium chrysogenum* and *A. nidulans* both of which produce penicillin and *Acremonium chrysogenum* producer of cephalosporin C. Despite the similarity of the penicillin and cephalosporin C pathways, *Penicillium* and *Acremonium* are taxonomically distant fungi belonging to the order *Eurotyales* and *Hypocreales,* respectively.

### 4.1 Mechanisms of LaeA and VeA regulation of penicillin biosynthesis in *Penicillium chrysogenum*

The *P. chrysogenum laeA* gene contains one intron and encodes a protein of 427 amino acids that shows 61% amino acids identity to the *A. nidulans* LaeA (Kosalková et al., 2009).

The *P. chrysogenum veA* homologous gene (called *velA*, according to the standard three letters gene designation used in most fungi other than *A. nidulans*) and the *laeA* gene have been cloned from the high penicillin-producing strain *P. chrysogenum* P2niaD18 (Hoff et al., 2010) and *laeA* has been also cloned from another high-producing strain, *P. chrysogenum* ASP-78, that contains at least five copies of the tandemly amplified penicillin gene cluster (Fierro et al., 1995). The two cloned genes are identical to that of the low producer parental strain *P. chrysogenum* Wis1255 (Kosalková et al., 2009).

**Figure 3:**
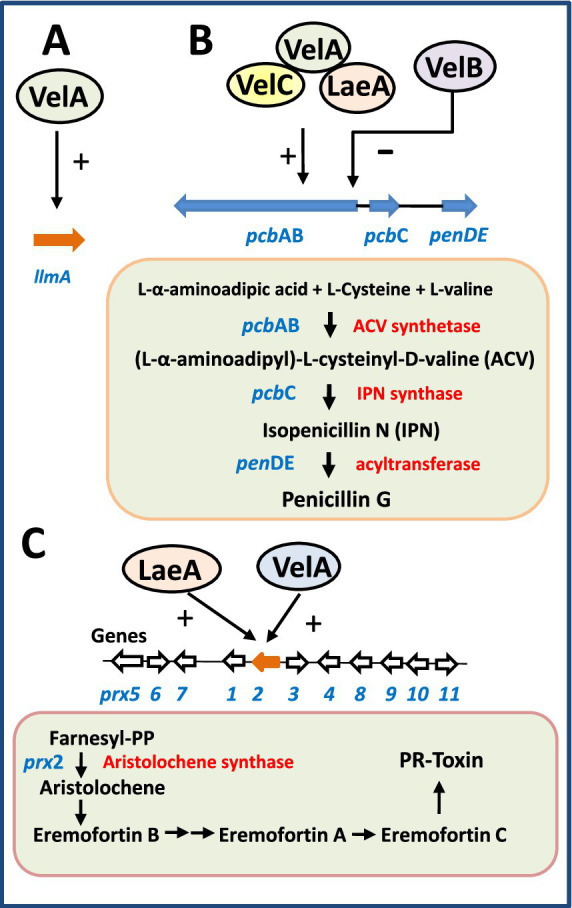
Scheme showing the mechanisms of control by components of the velvet complex in *P. chrysogenum*. The genes are shown in blue and the biosynthesis enzymes are labeled in red. **(A)** VelA positively controls expression of the gene *llmA* for a methyltransferase-like protein. **(B)** LaeA, VelA, and VelC control positively expression of genes encoding the three enzymes for penicillin biosynthesis, while VelB exerts a negative control on them. **(C)** LaeA and VelA, separately, positively control expression of the *prx2* gene encoding the aristolochene synthase. Seven genes in the *prx* cluster encoding the PR-toxin are silenced in *P. chrysogenum velA* or *laeA* mutants.

The LaeA regulatory effect is exerted at the transcriptional level of the *pcbAB, pcbC* and *penDE* genes encoding enzymes of the penicillin biosynthetic pathway (Kosalková et al., 2009; Figure 3). In addition, VelA is highly similar to *A. nidulans* VeA but does not conserve the canonical nuclear localization signal (NLS) and nuclear export signal (NES), features that may influence its interaction with VelB. Disruption of either *velA* or *laeA* in the high-producing strain drastically reduced penicillin biosynthesis, indicating that both proteins exert a positive regulatory effect on the expression of penicillin biosynthetic genes (Hoff et al., 2010). Both LaeA and VelA also influence sporulation in *P. chrysogenum*. In particular, *velA* mutants exhibited light-independent conidiation, whereas conidia formation in the parental strain was light-dependent (Hoff et al., 2010). Notably, transcriptional studies revealed that LaeA expression is increased in the *velA*-defective mutant demonstrating that there is a cross-regulation between these two proteins, i.e., VelA regulates negative *laeA* expression.

The velvet complex components VelC and VosA were later identified in *P. chrysogenum* (Kopke et al., 2013). Analyses of single and double knockout mutants revealed that different members of the velvet complex play distinct and sometimes opposing roles in conidia development and penicillin biosynthesis. For example, while LaeA positively regulates penicillin production, VelB acts as a negative regulator. Similarly, VelB and VosA promote conidiation, whereas VelC represses it (Kopke et al., 2013).

**Figure 4:**
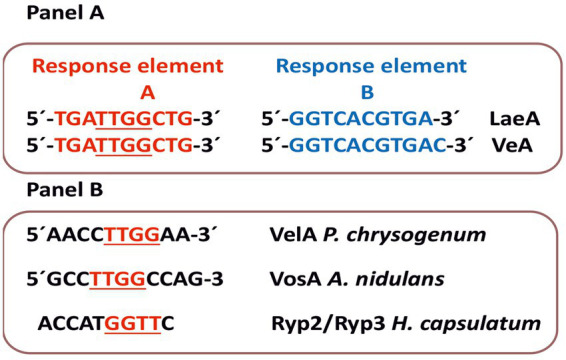
DNA sequences recognized by LaeA or velvet components. Panel **(A)** Above are shown the response element **A** (red) and **B** (blue) (Moon et al., 2023) recognized by LaeA. Below are shown the response elements A and B recognized by VeA. Panel **(B)** Eleven nucleotides DNA sequence of the VosA component of *A. nidulans*, the VelA component of *P. chrysogenum* and the Ryp2 (VosA)-Ryp3 (VelB) components of *H. capsulatum* as shown by electrophoretic mobility shift assays (EMSA) (Ahmed et al., 2013; Becker et al., 2016; Beyhan et al., 2013). Note that a TTGG sequence is common to the response elements A of Moon et al. (2023) and the sequences found by EMSA. (1) The sequence shown as response element B for VeA is the 5’ to 3’complementary sequence found by Moon et al. (2023) (the original is 5’GTCACGTGACCT3´). (2) The published sequence of Ryp2/Ryp3 binding does not indicate if the sequence is 5’ to 3’ (Beyhan et al., 2013).

Using *in silico* analysis and electrophoretic mobility shift assays (EMSA) of the VelA binding site of *P. chrysogenum*, Becker et al. (2016) identified a binding sequence in 46% of the promoters targeted by VelA (Figure 4). The VelA binding sequence found in *P. chrysogenum* and the VosA binding sequence in *A. nidulans* are similar to the binding sequence of the Ryp2 and Ryp3 components (50% nt identity in each case) of the velvet system of *Hystoplasma capsulatum* (Beyhan et al., 2013; Ahmed et al., 2013; Figure 4).

In summary, these findings suggest that the regulatory influence of LaeA on secondary metabolism and development in *P. chrysogenum*—including its role in penicillin gene expression—is mediated through interactions with the velvet complex, which specifically recognizes an 11-nucleotide specific sequence in the promoter of the target genes (Figure 4). Recently, gene regulatory networks controlled by VeA and LaeA have been described in *A. nidulans* (Moon et al., 2023). Using transcriptomic analysis, protein-DNA binding and protein–protein interactions studies it was shown that these two proteins control global networks that include many regulators such as VelB, VelC, AreA, MtkB and HogA.

These authors proposed that VeA and LaeA recognize two response element A and B with the sequences 5’TGATTGGCTG3’ and 5’GTCACGTGACCT3’ as sites to which they are bound interchangeably (Moon et al., 2023). Noteworthy, A sequences have some nucleotides in common with the VosA, VelB and Rye2/Rye3 binding sequences, particularly the core TTGG (Figure 4). The difference between the previously available binding site and the response element found by Moon et al. (2023) are perhaps due to the different analytical tools used in these studies where other proteins appear to interact *in vivo* with VeA or LaeA regulators.

### 4.2 The velvet complex in *Acremonium chrysogenum*: role of VeA in the control of cephalosporin C biosynthesis

Another interesting case of regulation by velvet components is the control of cephalosporin C biosynthesis in *A. chrysogenum*. Studies of the velvet complex in *A. chrysogenum* have focused on the VeA homologous (AcVeA) protein (Dreyer et al., 2007; Terfehr et al., 2017).

Using probes derived from conserved regions of *Neurospora crassa veA* gene, the Ac*veA* gene from *A. chrysogenum* was isolated by hybridization (Dreyer et al., 2007). The gene contains one intron and encodes a protein of 514 amino acids that shows moderate conservation, particularly in the N-terminal region, to VeA proteins belonging to fungi of the Hypocreales order and lower to those of Eurotyales order. The low conservation of the *veA* gene in different fungi indicates that it has evolved divergently in recent times from a common ancestor. The AcVeA protein contains a nuclear localization signal although this sequence differs from the NLS motifs identified in *Aspergillus* or *Penicillium*.

Disruption of *AcveA* resulted in an approximately 80% reduction in cephalosporin C production, similar to the effect observed for *veA* or *laeA* mutants in *P. chrysogenum* (see above). Complementation of the Ac*veA* mutant restored cephalosporin C production. Expression analyses showed that Ac*veA*-disrupted mutants exhibit significantly reduced transcription of the six genes involved in cephalosporin C biosynthesis (*pcbAB*, *pcbC*, *cefD1*, *cefD2*, *cefEF*, and *cefG*), with the *cefEF* gene—encoding the deacetoxycephalosporin C expandase/hydroxylase reduced by approximately 85%. The *cefEF* gene is located in the late cephalosporin C gene cluster (*cefEF–cefG*) and it is interesting that although the early and late gene clusters are located in different chromosomes (Gutiérrez et al., 1999) they are regulated by a common mechanism by AcVeA.

In a broader study examining the impact of AcVeA on secondary metabolism in different *A. chrysogenum* strains with distinct cephalosporin C production levels, approximately 50% of the secondary metabolite gene clusters were affected by *veA* disruption (Terfehr et al., 2017). The formation of most of them (89%) increased indicating that AcVeA primarily acts as a repressor of SM gene expression in *A. chrysogenum*. Phenotypic analyses further revealed that the mycelium of the Ac*veA*-disrupted mutant became fragmented earlier in the fermentation than in the parental strain and failed to form arthrospores (Bartoshevich et al., 1990; Martín and Demain, 2002).

#### 4.2.1 Does the *Acremonium chrysogenum* velvet complex contains the same components as the *Aspergillus* species?

The composition of the velvet complex varies among fungi. In *A. chrysogenum*, the LaeA homolog has not been experimentally characterized; bioinformatic analysis revealed that the putative LaeA protein is 55% identical throughout the entire protein sequence with the homologous protein of *Fusarium fujikuroi*. Notably, the VelC and VosA velvet components have not been detected in *A. chrysogenum*, and there is currently no information regarding interactions between VeA and LaeA within its velvet complex (Terfehr et al., 2017).

## 5 The velvet complex in *Fusarium* species

The role of LaeA in the regulation of diverse SM biosynthesis has been extensively studied in four *Fusarium* species: *F. fujikuroi*, *Fusarium verticillioides*, *F. graminearum*, and *Fusarium oxysporum*. Although these species synthesize distinct classes of SMs—e.g. gibberellins in *F. fujikuroi*, fumonisins in *F. verticillioides*, trichothecenes in *F. graminearum*, and various fusaric acid derivatives in *F. oxysporum*—in all cases, the biosynthetic pathways are under the regulatory control of the global regulator LaeA. To explore the conservation and characteristics of the VeA and LaeA regulators in fungi beyond the Eurotyales order, these components were characterized in *F. fujikuroi* and *F. verticillioides* (Butchko et al., 2012).

### 5.1 Fusarium fujikuroi

*Fusarium fujikuroi* (teleomorph *Gibberella fujikuroi*) is an agriculturally important fungus due to its ability to produce the plant growth factor gibberellin. In addition, this species synthesizes the pigments bikaverin and neurosporaxanthin, and several mycotoxins, such as fumonisins and fusarin C (Wiemann et al., 2010). The velvet complex components in *F. fujikuroi* are named FfVel1, FfLaeA1, and FfVel2, homologous to *A. nidulans* VeA, LaeA, and VelB, respectively (Wiemann et al., 2010). The amino acids sequence and protein localization studies revealed that FfLae1 and FfVel1 are nuclear proteins, as confirmed by fluorescent GFP fusion experiments. Yeast two-hybrid assays further confirmed that FfVel1 and FfLae1 physically interact, forming the velvet complex. All three characterized proteins—FfVel1, FfVel2, and FfLae1—affect the virulence of *F. fujikuroi* on rice plants (Wiemann et al., 2010).

The *Fflae1* gene encodes a 421-amino-acid protein that shares only 33% identity with *A. nidulans LaeA*, reflecting the taxonomic distance between *F. fujikuroi* and *A. nidulans*. Similarly, the Ff*vel1* gene of *F. fujikuroi* shows strong sequence similarity to homologous genes in *F. verticillioides* (91%) and *A. chrysogenum* (55%), while displaying lower identity with *Aspergillus* and *Penicillium* species. Despite the low sequence identity, the functional conservation of FfLae1 was demonstrated by complementation studies, e.g., the F*flae1* gene restored norsolorinic acid production (a precursor of sterigmatocystin) in *A. nidulans laeA*-deleted mutants and the *laeA* gene of *P. chrysogenum* complemented an F*flae1*-disrupted *F. fujikuroi* mutant.

The functional complementation of LaeA in different *laeA* defective mutants in taxonomically distant fungi, despite of the low overall similarity of these proteins, suggests that LaeA has evolved recently from an ancient common progenitor (see section 14).

Expression studies in *F. fujikuroi* Ff*vel1*-disrupted mutant indicate that hundreds of genes are differentially regulated, particularly those involved in transport systems, secondary metabolism biosynthesis, and differentiation. Among well-known SMs, FfVel1 positively regulates the production of fumonisin, gibberellins, and fusarin C, while negatively controls the biosynthesis of bikaverin (Wiemann et al., 2010).

Interestingly, in *F. fujikuroi* and also in other fungi, the expression of *laeA* is negatively regulated by Vel1 (Wiemann et al., 2010). The upregulation of Ff*lae1* expression in the Ff*vel1* mutant supports this regulatory mechanism and is consistent with similar findings in *A. nidulans* (Amaike and Keller, 2009) and *P. chrysogenum* (Hoff et al., 2010). This coordination ensures equilibrated expression of the velvet complex components as required at every time (Wiemann et al., 2010). The FfVel2 velvet component, homologous to VelB, exerts similar regulatory effects on SM biosynthesis and *F. fujikuroi* differentiation.

### 5.2 *Fusarium verticilloides* and *Fusarium graminearum*

*Fusarium verticillioides* (teleomorph *Gibberella moniliformis*) infects corn and is pathogenic to humans, being associated with the development of esophageal tumors. Several SMs produced by *F. fujikuroi* are also synthesized by *F. verticillioides*, including fumonisin, fusaric acid, fusarins, and the pigments aurofusarin and bikaverin (Brown et al., 2012a, 2012b).

Although *F. verticillioides* and *F. fujikuroi* share highly conserved SM gene clusters, the regulation of SM biosynthesis differs between these two species (Butchko et al., 2012). The *F. verticillioides* Fv*ve1* gene, was cloned and found to be highly similar to its *F. fujikuroi* counterpart (Myung et al., 2009). Strains disrupted in Fv*ve1* fail to synthesize fumonisin and fusarins, showing reduced expression of both the fumonisin structural and the regulatory gene *fum21.*

The Fv*lae1* gene was cloned to examinate the role of LaeA in this fungus (Butchko et al., 2012). Deletion of *Fvlae1* resulted in decreased expression of genes involved in the biosynthesis of fumonisin, bikaverin, fusaric acid, and fusarins. Interestingly, the regulatory pattern in *F. verticillioides* differs from that in *F. fujikuroi*; while these SMs are negatively regulated by LaeA1 in *F. fujikuroi*, they are positively regulated by LaeA in *F. verticillioides* (Butchko et al., 2012). Complementation of the Fv*lae1*-deleted mutant with the wild type allele restored fumonisin production, confirming the essential role of FvLae1 in SM biosynthesis.

Another velvet complex component, *F. verticilloides* FvVel2 (homologous to *A. nidulans* VelB), exhibits strong similarity to the corresponding VelB of *F. fujikuroi*. Expression of both Fv*ve1* and Fv*ve2* was impaired in the Fv*lae1* deletion mutant (Butchko et al., 2012).

In summary, these findings demonstrate that although the velvet proteins are conserved among related *Fusarium* species, the mechanism of action and regulatory interactions of specific velvet proteins can differ significantly. Notably, the *vosA* gene was not found in *F. verticillioides*, suggesting that this component may be absent in certain filamentous fungi (López-Berges et al., 2013).

*Fusarium graminearum* is an important pathogen of wheat and other cereals and produces numerous SMs, including trichothecenes, e.g., deoxynivalenol (DON), that are highly toxic to humans. The *F. graminearum* Fg*veA* gene encodes a 532-amino-acid protein that shares about 78% sequence identity with the VeA proteins of *F. verticillioides* and *F. fujikuroi*, while exhibiting lower identity to *A. nidulans* VeA (52%) (Merhej et al., 2012; Jiang et al., 2011). Deletion of Fg*veA* in *F. graminearum* leads to a reduction in aerial mycelium formation and surface hydrophobicity, while simultaneously increases conidiation. The Fg*veA*-disrupted mutant also displays a reduction in DON biosynthesis—approximately 21-fold lower than in the wild type strain—which correlates with decreased expression of the *TRI5* and *TRI6* genes involved in trichothecene biosynthesis. In addition, the mutant shows a reduction in the production of the pink pigment, associated with decreased expression of the PKS12 gene required for this pigment formation. Noteworthy, the Fg*veA* mutant of *F. graminearum* was found to be impaired in virulence on flowering wheat plants, a phenotype correlated with its reduced trichothecene production (Merhej et al., 2012).

Bioinformatic analyses revealed that, in addition to the VeA component, this fungus has genes homolog of VelB (Fg*velB*) and LaeA (Fg*laeA1*). The Fg*laeA1* gene encodes a 316-amino-acid protein, and deletion of this gene results in the loss of red pigment biosynthesis (Jiang et al., 2011).

Yeast two-hybrid analyses demonstrated that FgVeA does not interact with either FgVelB or FgLaeA1, providing the first documented instance of lack of VeA–VelB–LaeA interaction in filamentous fungi. Instead, FgVeA was shown to interact with six other proteins containing conserved methyltransferase domains. The individual deletion of each of the genes encoding these six methyltransferases had no detectable effect on virulence of *F. graminearum* (Jiang et al., 2011).

### 5.3 The velvet complex in *Fusarium oxysporum*, a pathogen common to plants and humans

*Fusarium oxysporum* infects more than a hundred different plant species, and can also cause fusariosis in immunocompromised humans (Dean et al., 2012). Its pathogenicity is mainly associated with the biosynthesis of the depsipeptide beauvericin and the production of the iron transporter ferrichrome, which interferes with iron homeostasis in infected hosts. Consequently, this fungus has been used as a model to compare the effects of the velvet complex on pathogenicity in both plants and animals. Among the four identified components of the velvet complex, VeA and LaeA are required for pathogenicity in both tomato plants and immunocompromised mice (López-Berges et al., 2013). Yeast two-hybrid analysis shows that there is an interaction between VeA-VelB, VeA-VelC, and VeA-LaeA. These results indicate that pathogenicity in both plants and animals is, at least in part, due to the interaction between VeA and LaeA.

No gene encoding a protein homologous to VosA was found in *F. oxysporum* as occurs also in other members of the *Fusarium* genus. If the VosA component is absent in this fungus, then binding of the complex to the target cannot be mediated by VosA and, therefore, other components of the velvet complex must recognize the binding sequence in the target genes.

In summary, the available evidence indicates that the interaction dynamics of the velvet complex components in *Fusarium* species are fundamentally distinct from those observed in members of the order Eurotyales (e.g., *Aspergillus* species). This divergence highlights the functional plasticity of the velvet regulatory system among filamentous fungi. The observed plasticity is probably due to changes in the velvet proteins that have been introduced in the late stages of fungal evolution in taxonomically related fungi. Taking into account the differences in the motifs and domains observed in the LaeA protein of different fungi (Supplementary Table 1) the information at the protein structure level suggests that domains other than the SAM binding motifs have evolved giving rise to differences in their amino acid sequences. This may explain the variable functional complementation of LaeA mutants with heterologous LaeA genes of related filamentous fungi. This functionality may not be conserved between Ascomycetes and Basidiomycetes although there is no experimental evidence about heterologous complementation between these two fungal classes.

## 6 LaeA regulation of peptaibols and other metabolites in *Thrichoderma* species

Peptaibols form a family of non-ribosomal synthesized peptides consisting of five to twenty amino acids, some of them non proteinogenic as it is the α-aminobutiric acid, and they usually contain an amino acid-alcohol at the C-terminal end (Marik et al., 2019; Reino et al., 2008). Peptaibols are important fungal metabolites because they have broad antimicrobial activity and also antitumor activity (Marik et al., 2019). These compounds are produced by several *Trichoderma* species and their regulation has been well studied in *T. reesei* and *Trichoderma longibrachiatum* (Shi et al., 2020; Aghcheh et al., 2013a, 2013b).

In the high peptaibols producer *T. longibrachiatum*, the *laeA* ortholog (Tl*laeA1*) was shown to control peptaibol biosynthesis. Deletion of Tl*laeA1* led to a strong reduction of conidia formation and peptaibols production; this regulation is exerted at the transcription level of *tlx1* and *tlx2*, two NRPS genes responsible for peptaibol synthesis. Conversely, constitutive expression of *TllaeA1* doubled peptaibol production, a feature of agricultural interest since *T. longibrachiatum* is used as biocontrol agent (Shi et al., 2020; Kubicek et al., 2019).

## 7 LaeA regulation of toxins in diverse fungi

Numerous SMs produced by filamentous fungi, known as mycotoxins, are toxic to plants or animals. Mycotoxins play an important role in communication between species since many of them are produced as defense against fungivore organisms. In addition, mycotoxins have sometimes antibacterial activities.

### 7.1 Toxins in *Alternaria alternata*

*Alternaria alternata* is a saprophytic fungus, that has the ability to infect various host plants by producing host-specific toxins (HSTs) (Tsuge et al., 2013; Wang et al., 2022). Seven different pathotypes of *A. alternata* have been described in the literature, including variants that infect Japanese pears, tomatos, apples, and strawberries (Imazaki et al., 2010; Takao et al., 2016). The toxins produced by these pathotypes are chemically distinct: the tomato pathotype synthesizes a polyketide toxin known as AAL, the apple pathotype produces cyclic peptide toxins referred to as AM toxins, and the strawberry pathotype generates a decatrienoic compound designated as AF toxin (Takao et al., 2016; Tsuge et al., 2013).

The AAL polyketide toxin produced by the tomato pathotype is encoded by a 13-gene cluster located on a conditionally dispensable chromosome of approximately 1.0 Mb (Akagi et al., 2009; Harimoto et al., 2007). Similarly, the AM and AF toxin gene clusters, found in the apple and strawberry pathotypes, respectively, are also located on dispensable chromosomes specific to each variant strain. In all cases, the cluster organization is highly conserved, comprising biosynthetic genes, transporter encoding genes involved in toxin secretion, and cluster-situated regulatory genes. The *laeA* gene was successfully cloned from the AAL-, AM-, and AF-producing pathotypes (Takao et al., 2016). The LaeA protein of *A. alternata* shares only 48% amino acid identity with that of *A. nidulans*, and it retains the characteristic SAM–binding domain.

Noteworthy, despite the structural diversity of the toxins, disruption of the *laeA* gene in each pathotype results in reduced growth, decreased sporulation, and complete loss of toxin production. Consequently, *laeA*-deficient mutants are non-pathogenic on their respective host plants. These findings collectively indicate that LaeA functions as a positive regulator of secondary metabolism, controlling expression of the toxin biosynthetic genes in each *A. alternata* pathotype (Takao et al., 2016).

### 7.2 LaeA controls pathogenicity in *Cochliobolus heterostrophus*

*Cochliobolus heterostrophus* is a necrotrophic fungus closely related to *A. alternata,* both of them belonging to the dothideomycete class. *C. heterostrophus* infects different cultivars of corn and caused devastating crop losses in last century due to the southern corn leave blight.

*Cochliobolus heterostrophus* produces a large number of SMs encoded by 25 PKS and 14 NRPS genes. Particularly important is the toxin T produced by the highly virulent *C. heterostrophus* T strain. The higher aggressivity of *C. heterostrophus* T as compared to the previous isolated *C. heterostrophus* O strain is due to the production of the host specific T-toxin. This compound is synthesized by two genes, *pks1* and *pks2,* that encode a family of lineal polyketides, and seven other genes encoding enzymes that modify the polyketides (Wu et al., 2012). These authors identified the genes homologous to *laeA* and *veA*, named Ch*lae1* and Ch*vel1,* and subsequently disrupted and overexpressed them*. S*tudies of these mutants demonstrated that both genes control positively expression of the T-toxin genes since the knockout mutant reduces drastically the biosynthesis of the T-toxin. In addition, mutations in Ch*laea1* or Ch*vel1* affect negatively the formation of 1,8-dihydroxynaphthalene (DHN)-melanin biosynthesis indicating that these two velvet proteins exert a negative control in melanization. In summary, ChLae1 and ChVel1 positively regulate T-toxin biosynthesis, pathogenicity and virulence, and negatively melanin biosynthesis and asexual differentiation in *C. heterostrophus*.

### 7.3 LaeA regulates positively PR-toxin biosynthesis in *Penicillium roqueforti* and *Penicillium chrysogenum*

The PR-toxin is a potent toxigenic compound produced during maturation of blue veined roquefort-type cheeses (Martín and Coton, 2016). Structurally the PR-toxin is a 15-carbon bicyclic sesquiterpene of the eremophilane class, produced mainly by *Penicillium roqueforti* and *P. chrysogenum*. The PR-toxin biosynthetic gene cluster and its encoded enzymes have been characterized in both species (Hidalgo et al., 2014). The first enzyme in the pathway, encoded by the *prx2* gene, is the aristolochene synthase, which catalyzes the cyclization of farnesyl diphosphate into aristolochene. This intermediate is subsequently converted into PR-toxin or other eremophilane-type metabolites (Figure 3).

During a study of global regulation by LaeA, *laeA* and *velA* deletion mutants were generated of a *P. chrysogenum* DSM strain lacking previous mutations in these loci (Veiga et al., 2012). Transcriptomic analyses in a chemostat culture under glucose limitation revealed that 23 genes were down-regulated in the *laeA* or *velA* mutants relative to the parental strain (Veiga et al., 2012). Notably, seven of these genes, including the aristolochene synthase gene, correspond to those forming the PR-toxin biosynthetic cluster (*prx2–prx8*) (Hidalgo et al., 2014; Martín, 2016).

In summary, the available evidence indicates that LaeA and VelA act as positive regulators of *P. chrysogenum* PR-toxin biosynthetic gene cluster, similarly to their regulatory role in the penicillin gene cluster (Figure 3).

### 7.4 Effect of LaeA on ochratoxin A biosynthesis

Ochratoxin A (OTA) is a mycotoxin produced by various species of *Aspergillus* and *Penicillium* with potential tumorigenic activity. It commonly contaminates agricultural products such as corn, barley, and wheat and may consequently be found in flour. In addition to cereals, OTA contamination has been reported in coffee beans, dried fruits, legumes, and other food products.

Structurally, OTA is a hybrid molecule composed of a chlorinated polyketide dihydroisocoumarin moiety linked via an amide bond to the amino acid phenylalanine. The isocoumarin moiety is a tetraketide derived from the condensation of four acetate units, with an additional carbon atom at position C-7 originated from methionine (O’Callaghan et al., 2013).

Recent genomic studies have identified the gene cluster responsible for OTA biosynthesis in six filamentous fungi, including five *Aspergillus* species and *Penicillium nordicum*. This cluster is highly conserved across species and comprises the core genes *otaA*, *otaB*, *otaC*, *otaR*, and *otaD*. Ferrara et al. (2021) further analyzed this cluster and discovered a small open reading frame encoding a 120 amino acid protein located between *otaA* and *otaB*. Functional inactivation of this gene, named *otaY*, using CRISPR/Cas9 technology demonstrated that it encodes a polyketide cyclase involved in the cyclization of the tetraketide intermediate during the synthesis of the isocoumarin moiety.

#### 7.4.1 Ochratoxin A biosynthesis in *Aspergillus carbonarius* and *Aspergillus niger*

*Aspergillus carbonarius* produces ochratoxin A, a metabolite with strong insecticidal activity thought to function as a fungal defense mechanism against fungivorous insects (Wang et al., 2016; Crespo-Sempere et al., 2013; Linde et al., 2016). Deletion of the global regulatory gene *laeA* in *A. carbonarius* results in a significant reduction (68–97%) of OTA biosynthesis (Crespo-Sempere et al., 2013).

More recently, the *laeA* gene was characterized in an ochratoxigenic strain of *A. niger*. The gene contains one intron and encodes a 375-amino-acid protein that conserves the SAM binding motif and shares approximately 75% identity with *laeA* homologs in other *Aspergillus* species, (Zhang X. et al., 2022). Functional analysis using *laeA*-disrupted and *laeA*-overexpressing strains revealed that the disruption of *laeA* led to increased conidiation, whereas overexpression reduced the formation of asexual conidia. This alteration may reflect either a deficiency of *laeA* or indirect effects on the expression of other developmental regulators, such as *brlA*.

Disruption of *laeA* also reduced OTA production in *A. niger*, consistent with observations in *A. carbonarius*, indicating that *laeA* acts as a positive regulator of OTA biosynthesis. However, *laeA* overexpression in *A. niger* did not lead to increased OTA levels, probably due to indirect effects of *laeA* on competing metabolic pathways that deprive the strain of precursors or intermediates required for OTA synthesis. Quantitative RT-PCR analyses confirmed that all five core genes of the *ota* biosynthetic cluster were down-regulated in the *laeA*-deficient mutant, while four of them (with the exception of the P450 monooxygenase gene) were up-regulated in the *laeA*-overexpressing strain.

In addition, the *veA* gene of the ochratoxigenic *A. niger* has been deleted to assess its role in secondary metabolism and development. The *veA* gene contains two introns and encodes a 555-amino-acids protein. Deletion studies revealed that VeA functions as a positive regulator of conidiation, OTA biosynthesis and oxidative stress response, independently of light or dark conditions (Wang et al., 2019). Deletion of *veA* nearly abolished OTA production and markedly reduced expression of the *otaKS* gene, which encodes the polyketide synthase responsible for forming the tetraketide intermediate of OTA biosynthesis.

Further studies in *A. niger* are needed to elucidate the molecular interaction between LaeA and VeA in the coordinated regulation with morphological differentiation, OTA biosynthesis, and oxidative stress responses.

#### 7.4.2 Aspergillus ochraceus

*Aspergillus ochraceus* is a well-known producer of OTA, particularly after infecting cereals, coffee beans, and dried fruits. Genomic analyses have revealed that this species contains the complete *ota* biosynthetic cluster.

LaeA, VelA, and VelB drastically affect growth, sporulation, and OTA formation in *Aspergillus ochraceus* (Wang et al., 2019; Zhang et al., 2018). An *A. ochraceus laeA*-defective mutant exhibited a marked reduction in conidia formation under dark conditions, along with a significant decrease in OTA production. This reduction is associated with the down-regulation of *ota* biosynthetic genes (Wang et al., 2019). Additionally, the deletion mutants showed a diminished ability to infect pears. However, since the *laeA* mutation affects the expression of approximately 66% of genes, other protein factors beyond *laeA* likely contribute to the reduced pathogenicity.

## 8 LaeA regulation of statins biosynthesis in several fungi

Statins are inhibitors of cholesterol biosynthesis and are pharmacologically important anticholesterolemic agents. They act by inhibiting 3-hydroxy-3-methylglutaryl-CoA (HMG-CoA) reductase, a key enzyme in the mevalonate pathway responsible for producing mevalonic acid, a direct precursor of cholesterol formation. Ecologically this mode of action indicates that some fungi synthesize statins as a mechanism of defense against animals that contain cholesterol in the membranes. The statins do not act against fungi that contain ergosterol instead of cholesterol. This inhibition of cholesterol biosynthesis may protect fungi against predatory animals.

There is evidence that LaeA regulates statins biosynthesis in several fungi, including lovastatin in *Aspergillus terreus*, mevastatin in *Penicillium citrinum*, and compactin in *Monascus pilosus*, although experimental evidence remains limited in all cases (Bok and Keller, 2004; Xing et al., 2010; Zhang and Miyake, 2009). The expression of lovastatin biosynthetic genes subcloned in *A. nidulans* was significantly up-regulated. Moreover, introduction of the *A. nidulans laeA* gene into *A. terreus* resulted in two to seven-fold increase in lovastatin production (Bok and Keller, 2004). However, no detailed studies have been made on the characterization of the *laeA* gene in *A. terreus* or to elucidate the precise mechanism of lovastatin biosynthesis LaeA regulation.

### 8.1 Monacolin K in *Monascus pilosus*

An up-regulated transcript sequence tag in an improved monacolin producing *M. pilosus* mutant was isolated using a subtractive hybridization technique, and it corresponded to the *laeA* gene (Zhang and Miyake, 2009). The cloned gene showed 70% nucleotide identity with *A. nidulans laeA*, and the predicted protein contained the conserved SAM binding sequence. Interestingly, a single amplification band was obtained from *M. pilosus* genomic DNA, whereas two distinct bands were amplified from cDNA under identical PCR conditions. These two RT-PCR products differed by 270 nucleotides. The *laeA* gene in *M. pilosus* contains two introns, of 270 and 70 nucleotides, respectively, and the two cDNA bands result from alternative splicing of the 270-nt intron in the transcript. Splicing of this intron allows the formation of a small ORF encoding a protein of 299 amino acids. However, it is unclear if the small LaeA form encoded in this ORF has the same regulatory properties as the full-size protein. The relative abundance of the long and short transcript forms varies between the growth phase and late production stage, and is also influenced by nutrient conditions. Antisense-mediated silencing of the *M. pilosus laeA* gene resulted in decreased expression of the monacolin biosynthetic genes. Although introns have been reported in *laeA* genes from various fungi, this was the first example suggesting that alternative splicing may affect the transcriptional control of *laeA* expression (Zhang and Miyake, 2009).

### 8.2 LaeA in *Penicillium citrinum* and the control of mevastatin biosynthesis

Mevastatin (compactin) is a potent statin clinically used in the treatment of hypercholesterolemia. The *Penicillium citrinum laeA* gene was cloned by PCR amplification using primers designed from conserved regions flanking *laeA* sequences in other fungi (Xing et al., 2010). The gene contains a 56 bp intron and encodes a 427-amino-acid protein that shares 95% identity with the LaeA protein of *P. chrysogenum*. The *P. citrinum* LaeA protein includes a conserved SAM binding motif, although it has small variations compared to that of typical methyltransferases. Mevastatin biosynthesis in *P. citrinum* was proposed to be regulated by the transcriptional factor MclR, which binds asymmetric repeat sequences present in several genes involved in mevastatin biosynthesis. Noteworthy, a putative MclR recognition sequence was also identified in the promoter region of the *P. citrinum laeA* gene, suggesting that MclR may regulate *laeA* expression (Xing et al., 2010). This regulatory relationship resembles the control of *laeA* transcription by the AflR regulator in sterigmatocystin-producing fungi (Bok and Keller, 2004). It has been proposed that the *laeA* gene interacts with an MclR-like regulatory protein, representing a potential mechanism for coordinated regulation of LaeA-dependent SM biosynthesis (Xing et al., 2010). However, detailed mechanistic studies on *laeA*-mediated control of mevastatin biosynthesis in *P. citrinum* are still lacking, and further work is needed to elucidate the precise interactions between *laeA* and MclR.

## 9 Regulation of pigments by LaeA

Another example of regulation by the nuclear regulator LaeA involves the control of pigments in different fungi. Species of *Monascus* produce at least six azaphilone (red, orange or yellow) pigments and citrinin, a nephrotoxic compound (Campoy et al., 2005). Natural pigments occur in many plants and in some fungi; these pigments serve ecological functions to attract insects that contribute to plants seed or fungal spore dispersion. Pigments production during *Monascus* growth on rice are used to prepare red rice, a common food in east Asian countries.

In a *Monascus ruber* mutant deficient in *laeA*, production of the six characteristic *Monascus* pigments and citrinin was markedly reduced. This mutant also displayed enhanced aerial mycelium and conidiation but was unable to form ascospores (Liu et al., 2016). Complementation with the *laeA* gene restored normal conidiation and ascospore formation, confirming the regulatory role of LaeA. Despite these findings, the molecular mechanisms underlying LaeA-mediated regulation of pigment and citrinin biosynthesis in *M. ruber* remain largely unresolved.

Another important group of pigments are carotenoids. Light induction of carotenoids biosynthesis in some fungi is well known (Castrillo et al., 2018). The velvet proteins Ve1 (VeA), Vel2 (VelB), and VosA, as well as LaeA have been identified in the model organism *N. crassa* that belongs to the Sordariomycetes class (Sarikaya-Bayram et al., 2019). These components form two different complexes *in vivo*, the trimeric complex Vel1-Vel2-LaeA and the dimer Vel2-VosA. The four components show a light independent nucleocytoplasmic localization. The Ve1 and Ve2 components of *N crassa* are similar to those of *A. nidulans* and, indeed, heterologous complementation of *A. nidulans* mutants was observed whereas LaeA and VosA are clearly different and no heterologous complementation of *A. nidulans* mutants was observed. The heterotrimeric Ve1-Ve2-LaeA complex enhances sexual reproduction, represses conidia formation and supresses siderophore formation under iron limiting conditions. Studies with velvet component deleted mutants showed a significant reduction of carotenoid biosynthesis under light inducing conditions in the Ve1 and Ve2 mutant and a partial reduction in the LaeA mutant, indicating that these components exert a positive regulation of carotenoid biosynthesis (Sarikaya-Bayram et al., 2019).

## 10 LaeA regulation of citric acid secretion in several *Aspergillus* species

Beyond its well-established role in regulating secondary metabolism in fungi, LaeA has also been implicated in the control of citric acid production in several industrially important fungi, including *A. niger*, *A. carbonarius*, and the koji mold *Aspergillus luchuensis*. Citric acid is properly speaking a primary metabolite, since it is essential in aerobic fungi as a component of the tricarboxylic acid cycle. However, some fungi, such as *Aspergillus*, secretes large amounts of citric acid and this is used for the industrial production of this organic acid. Both SM and the secreted citric acid are formed during the late phase of the culture after the rapid growth phase when most vegetative growth has already occurred.

In natural environments, acidification of the substrate through citric acid secretion by *A. niger* and *A. carbonarius* provides a competitive advantage by suppressing bacterial growth. The link of LaeA to citric acid secretion was obtained by the characterization of a non-acidifying mutant of *A. niger* that was defective in citric acid excretion (Niu et al., 2015). Using bulk segregant analysis combined with high-throughput sequencing, the mutation responsible for the non-acidifying phenotype was mapped to the *laeA* gene. Complementation of this mutant with the wild type *laeA* allele from the parental strain fully restored citric acid production, whereas targeted deletion of *laeA* in *A. niger* completely abolished citric acid secretion. These results are consistent with previous observations in *Aspergillus oryzae*, where *laeA* was shown to be essential for kojic acid production (Oda et al., 2011). Similarly, in an *A. carbonarius laeA* deletion mutant, citric acid secretion decreased by 74–96%.

Recently, a LaeA regulated *cexA* gene from *A. niger*, responsible for citric acid secretion, was independently cloned by two research groups (Steiger et al., 2019; Odoni et al., 2019). CexA is a transporter member of the major facilitator superfamily (MFS, subclass DHA1). Deletion of *cexA* results in a complete loss of citric acid secretion, confirming that CexA serves as the principal citric acid exporter in these fungi. Heterologous expression of *cexA* in *Saccharomyces cerevisiae* enabled the yeast to excrete citric acid, and gene duplication of *cexA* in *A. niger* significantly enhanced citric acid production.

More recently, the role of LaeA in regulating citric acid secretion in *A. luchuensis* has been elucidated (Kadooka et al., 2020). *A. luchuensis*, a member of the black *Aspergilli* group, is traditionally used in the fermentation of shochu, a Japanese distilled spirit. Both *A. luchuensis* and its albino mutant (*A. kawachii*) are utilized in shochu production, where citric acid secretion prevents bacterial contamination during fermentation. Using analysis of gene expression, along with gene disruption and complementation experiments, it was demonstrated that the silenced gene in the *laeA*-deficient strain corresponded to the citric acid transporter gene *cexA* (Kadooka et al., 2020). Complementation of the mutant with the wild type *cexA* allele restored citric acid secretion to wild type levels. Moreover, chromatin immunoprecipitation followed by quantitative PCR (ChIP–qPCR) analysis confirmed that in *A. luchuensi*s LaeA regulates citric acid production in parallel to epigenetic control of *cexA* expression, specifically by modulating histone modifications at H3K4 and H3K9 (Kadooka et al., 2020). The CexA protein contains 12 transmembrane domains and is proposed to localize primarily to the plasma membrane. However, the complexity of the fungal secretory system, which involves multivesicular transport pathways, suggests that CexA may also associate with membrane vesicles (Martín and Liras, 2024).

Membrane transporters of the MFS class, containing 12 transmembrane domains, have also been identified in several of the SM gene clusters. e.g., in *A. chrysogenum* cephalosporin gene cluster (Teijeira et al., 2009; Ullán et al., 2002; Ullan et al., 2010). Genome wide analysis of transporters encoded in SM gene clusters provided scattered evidence showing that the response to LaeA affects expression of these transport systems; however, detail analysis of the substrate selectivity of these transporters is still lacking. These observations agree with the finding that the citric acid exporter in *A. luchuensis* is also an MFS class transporter protein.

## 11 Regulation by LaeA in Basidiomycetes: LaeA control of siderophores and antitumor compounds

The role of LaeA in regulating SM biosynthetic pathways and developmental processes in ascomycetous is well known but its regulatory functions in Basidiomycetes remain poorly understood.

**Figure 5:**
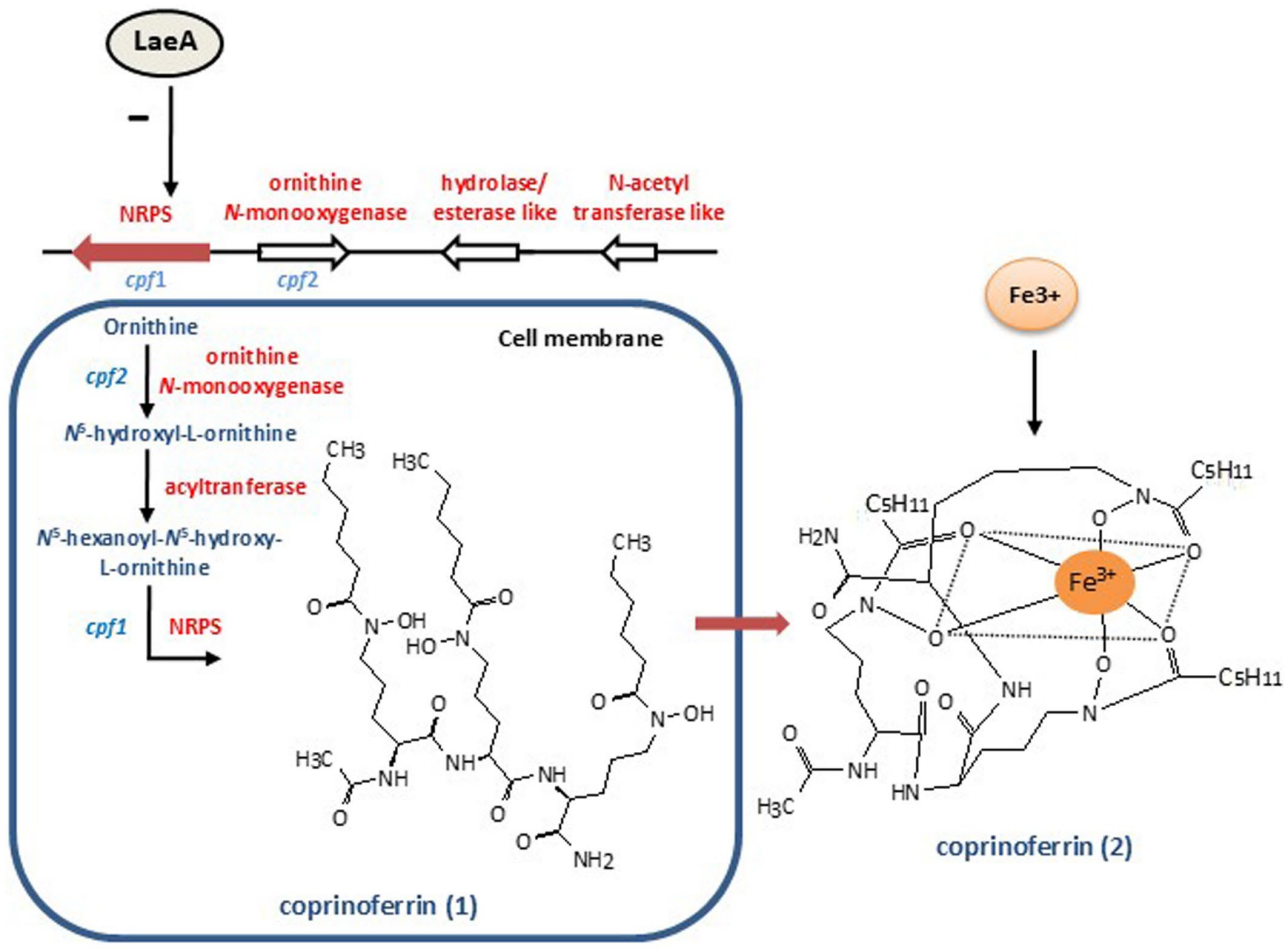
Negative control by LaeA of coprinoferrin biosynthesis in the Basidiomycete *Coprinopsis cinerea*. The coprinoferrin gene cluster is shown above; only genes *cpf1* and *cpf2*, have been genetically characterized. The coprinoferrin biosynthesis pathway is shown below indicating in blue the genes and in red the enzymes. The cellular lineal form of coprinoferrin (1) and the extracellular cyclic form, coprinoferrin (2) are shown.

Recent studies have shown that LaeA also regulates the biosynthesis of SMs in Basidiomycetes, including the siderophore coprinoferrin in *Coprinopsis cinerea* and the triterpenoid ganoderic acid in *Ganoderma lingzhi.* A knockout mutant of the *laeA* gene in *C. cinerea*, led to the production of a novel compound named coprinoferrin (Tsunematsu et al., 2019). These findings indicate that LaeA negatively regulates the biosynthesis of this siderophore. Chemical characterization revealed that this compound consists of three identical hexanoyl-N-hydroxyornithine units synthesized by a nonribosomal peptide synthetase (NPS2) (Tsunematsu et al., 2019) (Figure 5). This structure is similar to that of fusarinin C in Ascomycetes, that contains two repeated unit of anhydromevalonyl-N-hydroxyornithine (Hai et al., 2020). The presence of hydroxyornithine within the coprinoferrin structure is consistent with previous reports describing this fragment as a key iron-chelating moiety in siderophores from various Ascomycetes (Liras and Martín, 2023). The identified NRPS was responsible for coprinoferrin biosynthesis, as deletion of the corresponding NRPS gene abolished coprinoferrin production. Furthermore, the NPS2-deficient mutant exhibited stunted hyphal growth and failed to form fruiting bodies, highlighting the importance of the siderophore in *C. cinerea* development.

Molecular analysis of the coprinoferrin biosynthesis revealed a gene cluster containing a NRPS gene located adjacent to an ornithine hydroxylase encoding gene in a region of 17 conserved genes. The identified NRPS features a single adenylation (A) domain and tandemly triplicated thiolation (T)–condensation (C) didomains, indicating that it represents a new class of iterative NRPSs (class VI) common to Basidiomycetes (Bushley et al., 2008; Brandenburger et al., 2017). Gene expression analyses further revealed that *laeA* is moderately expressed during the developmental stages of *C. cinerea*, particularly in the mycelial phase, where it acts as a negative regulator of siderophore biosynthetic genes prior to fruiting bodies development thus controlling the transition from mycelium to fruiting bodies (Muraguchi et al., 2015; Tsunematsu et al., 2019). The importance of LaeA control of siderophore in Basidiomycetes is in agreement with previous results in Ascomycetes where it is known that LaeA also controls biosynthesis of siderophores (Perrin et al., 2007).

Siderophores are properly speaking primary metabolites since they are essential for iron transport and metabolism in all fungi. The role of LaeA on siderophore biosynthesis has been studied in *A. fumigatus* but there is little information on the control by LaeA of siderophores in many other filamentous fungi (Perrin et al., 2007). LaeA regulation of siderophore biosynthesis is also important in *F. oxysporum,* a pathogen of plants and immunocompromised mice. In this fungus the effect of LaeA is due to the modulation of the biosynthesis of the ferrichrome siderophore that interferes with iron nutrition in the infected hosts (López-Berges et al., 2013).

### 11.1 Ganoderic acid, an antitumor compound controlled by LaeA

Recent studies in Basidiomycetes have increasingly focused on the regulation of triterpene biosynthesis, particularly squalene-derived antitumor compounds such as ganoderic acid, produced by *Ganoderma lingzhi* and clavaric acid, produced by *Hypholoma sublateritium* (Godio et al., 2007; Luo et al., 2022). Mushrooms are prolific producers of terpenoids, many of which have been used for centuries in traditional medicine across East Asia. Various ganoderic acids produced by *Ganoderma* species have been shown to induce apoptosis in cancer cells, inhibit lung cancer metastasis, and enhance antiviral activity against human immunodeficiency virus HIV (Tang et al., 2006; Chen et al., 2008; García-Estrada et al., 2025). The biosynthetic pathways of these triterpenoids have been extensively characterized by several research groups (Godio et al., 2007; Godio and Martín, 2009; Chen et al., 2012; Ahmad et al., 2022). Ganoderic acid derives from squalene via cyclization into lanosterol, followed by specific oxidative and structural modifications.

The global regulator LaeA has been shown to play a crucial role in controlling ganoderic acid biosynthesis in *Ganoderma lingzhi*. Deletion of the *laeA* gene, identified within the genome of this fungus, resulted in a dramatic reduction in the production of ganoderic acid and its biosynthetic intermediates (Luo et al., 2022). The *laeA*-deficient mutant also exhibited reduced formation of asexual spores. Additionally, overexpression of *laeA* significantly enhanced ganoderic acid production, demonstrating that LaeA acts as a positive regulator of this antitumor triterpenoid.

Quantitative RT–PCR analyses of *laeA*-deleted mutants revealed a drastic reduction in expression of genes involved in the conversion of squalene into ganoderic acid. Despite these insights, our understanding of LaeA function in Basidiomycetes remains limited. Basidiomycetes generally do not have a canonical velvet complex, though they may have distantly related regulatory proteins. Nevertheless, the availability of several Basidiomycete genome sequences showing the presence of *laeA* homologs suggests that LaeA likely serves a broader regulatory role in controlling the biosynthesis of additional SMs across this fungal class.

## 12 Role of LaeA in cellulose degradation in different fungi

The biodegradation of lignocellulose by fungi is of critical importance for production of biofuels. This process has been extensively investigated in cellulolytic and mycoparasitic fungi, including *Trichoderma atroviride* (Aghcheh et al., 2013a), *T. reesei* (Aghcheh et al., 2013b; Seiboth et al., 2012), *Penicillium oxalicum* (Li et al., 2016; Zhang et al., 2020), and *Myceliophthora thermophila* (Zhao et al., 2023). The glucose released from the degradation of the *β*-1,4-linked polysaccharide hemicellulose provides a renewable carbon source that supports the growth and metabolic activity of these filamentous fungi.

The *laeA* gene of *Trichoderma reesei* was identified through genome mining and bioinformatic comparison with *laeA* genes from fungi of diverse taxonomic classes (Martínez et al., 2008; Seiboth et al., 2012). In a *laeA*-deficient mutant of *T. reesei*, expression of seven cellulase-encoding genes was abolished, and transcription of β-xylosidase and xylanase genes was also significantly repressed. Reintroduction of the *laeA* gene restored cellulase gene expression (Seiboth et al., 2012). Interestingly, the *T. reesei laeA* gene cannot complement a *laeA*-defective mutant of *A. nidulans*, but it fully complements its own *laeA* mutant, indicating a degree of species-specific functional divergence. Yeast two-hybrid analyses revealed that the *T. reesei* LaeA protein does not interact with the VeA component from *A. nidulans*, but it interacts with its own VeA homolog. Moreover, LaeA is required for expression of the *veA* homolog in *T. reesei*, but it does not regulate the expression of other components of the velvet complex (Aghcheh et al., 2013a, 2013b).

In *T. reesei*, cellulase gene expression is also regulated by the transcription factor Xyr1, and interestingly, LaeA controls expression of the *xyr1* gene. Chromatin immunoprecipitation (ChIP) analyses targeting histone methylation marks (H3K4me3 and H3K9me3) associated with transcriptionally active or condensed chromatin revealed that 4,089 genes carried such methylation signatures, among which 75 genes were identified as being directly regulated by LaeA (Aghcheh et al., 2013b).

The cellulolytic system of *P. oxalicum* (syn. *P. decumbens*) has been extensively characterized at the molecular level. This filamentous fungus produces a broad spectrum of cellulases, cellobiose hydrolases, and other lignocellulose-degrading enzymes, whose expression is tightly regulated by multiple transcription factors (Li et al., 2013). Over the past two decades, *P. oxalicum* has been intensively studied in China and utilized for industrial cellulase production. Transcriptional control of its cellulolytic enzymes involves at least 20 regulatory proteins, including LaeA, ClrB, CreA, XlnR, Ace1, and AmyR. Gene disruption studies have shown that these regulators can function as either activators or repressors, coordinating enzyme expression in a synergistic and dose-dependent manner (Li et al., 2015). Among them, the global regulator LaeA plays a particularly important role, positively influencing the expression of most cellulolytic enzymes.

Analysis of *laeA*-deleted mutants in combination with either inactivation or overexpression of major transcription factors revealed that LaeA functions independently of these regulators. Interestingly, LaeA also exerts a negative regulatory effect on β-xylosidase expression and secretion; consequently, *laeA*-deficient mutants overproduce xylosidases, particularly when positive transcription factors are simultaneously overexpressed (Li et al., 2016).

In *Myceliophthora thermophila* (syn. *Sporotrichum thermophilum*), a thermophilic fungus with strong cellulolytic activity, LaeA plays a key regulatory role in the formation of cellulolytic enzymes. This fungus has been developed as a fungal platform for the industrial production of cellulolytic enzymes used in biofuel generation (Li et al., 2020). *M. thermophila* exhibits an optimal growth temperature of approximately 45 °C, making it an attractive host for high-temperature fermentation processes. However, its relatively slow growth rate limits its industrial potential, and improved carbohydrate utilization is therefore desirable. Interestingly, the putative methyltransferase LaeA regulates both growth rate and sugar metabolism in this fungus. A *M. thermophila* mutant lacking *laeA* displays increased growth rate and enhanced cellulolytic activity when cultivated on cellulose. LaeA appears to control in this fungus the expression of several negative transcriptional regulators, including Grf-1, Grf-2, Grf-3, and the global regulator CreA. In addition, LaeA influences glucose transport and the expression of key metabolic enzymes such as phosphoenolpyruvate carboxykinase and other gluconeogenic enzymes, leading to increased biomass accumulation and glucose utilization (Zhao et al., 2023).

## 13 Other interacting partners of the velvet complex: LaeA-like methyltransferases. Do they control the biosynthesis of secondary metabolites?

Several LaeA-like methyltransferases (Llm) have been identified in different filamentous fungi. An important question is whether these Llm proteins interact with VeA or other velvet components in the regulation of SM biosynthesis and cellular differentiation. In *A. nidulans*, nine Llm proteins have been identified, all of which contain the SAM binding motif characteristic of methyltransferases. Functional analysis through gene disruption revealed that only one of these genes, *llmF*, significantly affects the regulation of sterigmatocystin biosynthesis (Palmer et al., 2013).

The *llmF* deletion mutant exhibits increased sterigmatocystin production, indicating that LlmF acts as a negative regulator of this mycotoxin biosynthesis. LlmF displays a dual cytoplasmic and nuclear distribution in contrast to LaeA, that is mainly located in the nucleus (Bayram et al., 2015). Mechanistically, LlmF interferes with the transport of VeA into the nucleus. In the *llmF* mutant, VeA accumulates in the nucleus, whereas overexpression of LlmF results in a reduced nuclear concentration of VeA. Although LlmF physically interacts with VeA, no direct methylation of VeA has been observed *in vitro*.

Further insights into LlmF-related regulatory mechanisms in *A. nidulans* were provided by the discovery of a membrane-bound heterotrimeric complex, VapA–VipC–VapB. In this complex, VapA is a FYVE like zinc finger protein located in the plasma membrane, while VipC (also known as LlmB) and VapB are small methyltransferases that form a dimer. VapA acts as a sensor protein, capturing and retaining the VipC–VapB dimer at the membrane until suitable extracellular environmental signals are detected. Upon activation, the heterotrimeric complex releases the VipC–VapB dimer into the cytosol, from where it translocates into the nucleus. The dimer interacts with VeA, thereby limiting its nuclear accumulation in the free form (Sarikaya-Bayram et al., 2014). This interaction consequently reduces the formation of the VeA–VelB–LaeA regulatory complex.

In summary, in *A. nidulans* the central component of the velvet complex, VeA, interacts with four putative methyltransferases: LaeA, LlmF, VipC, and VapB. This observation suggests that VeA contains a specific interaction domain for LlmF binding. A similar interaction domain has been identified in the C-terminal region of LaeA (Bayram et al., 2008); however, corresponding domains in the other proteins of the multimeric complex have not yet been characterized.

### 13.1 The Llm1 methyltransferase in *Cochliobolus heterostrophus*

A LaeA-like methyltransferase, designated Llm1, functionally similar to LlmF, has been studied in the T-toxin–producing fungus *C. heterostrophus*. Interestingly, the regulatory mechanism of Llm1 in *C. heterostrophus* differs from that of LlmF in *A. nidulans*. Deletion of the *llm1* gene leads to increased T-toxin production, indicating that Llm1 acts as a negative regulator of T-toxin biosynthetic gene expression (Bi et al., 2013). In this species, regulation of T-toxin biosynthesis occurs through cytosolic sequestration of VeA together with Llm1, that limits VeA nuclear translocation and thereby reduces its transcriptional regulatory activity.

### 13.2 LaeA-like methyltransferases in *Penicillium chrysogenum*

LaeA-like proteins have also been identified in the penicillin producer *P. chrysogenum*, where the VeA homolog, VelA, interacts with at least seven Llm proteins (Becker et al., 2016). Using chromatin immunoprecipitation followed by DNA sequencing (ChIP-seq), these authors identified numerous VelA target genes across the *P. chrysogenum* genome, including genes involved in secondary metabolism and cell differentiation. Among these targets were seven genes encoding putative methyltransferases containing the conserved SAM–binding motif.

One of these methyltransferases, PcLlmA, regulates penicillin biosynthesis and was shown—through yeast two-hybrid analysis—to interact directly with the VelA component of the velvet complex. Protein localization studies demonstrated that both interacting partners co-migrate to the nucleus, suggesting their cooperation in nuclear regulatory processes. This interaction plays a regulatory role in both penicillin biosynthesis and conidiation. Functional analysis of *llmA* mutants, complemented with the wild type allele, as well as overexpression strains, revealed that LlmA increases conidia formation. Although *llmA* overexpression did not directly alter penicillin biosynthetic gene expression, LlmA was found to affect spore germination and pellet morphology, that may explain the increase in penicillin production in submerged liquid cultures.

### 13.3 The *Penicillium oxalicum* LlmMTR23B protein controls polysacharides hydrolases and secondary metabolites biosynthesis

In a genome-wide search for LaeA-like methyltransferase genes 11 putative methyltransferase encoding genes containing the MTR23 domain (Finn et al., 2016), in addition to *laeA*, were identified in *P. oxalicum* (Zhang et al., 2020). Among these, only three genes—*laeA* and *llm-mtr23B*—were found to be actively expressed across different growth stages, whereas the remaining nine *llm* exhibited low or negligible expression levels.

Expression profiling using *P. oxalicum* mutants deleted for either *laeA* or *mtr23B* showed that transcription of *mtr23B* is independent of *laeA*, and conversely, expression of *laeA* is not influenced by *mtr23B*.

Comparative genomic analyses further showed that the *mtr23B* gene is conserved among many Ascomycetes, particularly within the *Penicillium* genus. These findings indicate that Mtr23B represents a distinct class of methyltransferases differing from LaeA both in structure and regulatory role. The Mtr23B protein in *P. oxalicum* is involved in the regulation of 10 physically linked genomic regions corresponding to SM gene clusters. Two of these regions include: (1) a cluster responsible for the dihydroxynaphthalene-melanin biosynthetic pathway, and (2) a cluster containing genes encoding enzymes for the biosynthesis of roquefortine–meleagrin (García-Estrada et al., 2011; Martín and Liras, 2015).

The remaining eight SM gene clusters are distributed across five of the eight chromosomes of *P. oxalicum*. Notably, a critical set of Mtr23B-regulated enzymes is involved in lignocellulose utilization, that are important for industrial applications (Zhang et al., 2020). How the expression of these gene clusters is coordinated and whether it involves epigenetic chromatin rearrangement remains an open question.

### 13.4 Is expression of silent *llm* genes limiting for their biological activity?

An interesting finding emerged from the study of *llmG*, one of the *LaeA*-like methyltransferase encoding genes in *A. nidulans* (Palmer et al., 2013). Expression of *llmG* is negatively regulated by the global transcriptional repressor McrA (Oakley et al., 2017; Grau et al., 2019). Because *llmG* expression is normally repressed by the global regulator McrA, its effect on secondary metabolism and fungal differentiation is likely to be limited in the wild type. Overexpression of *llmG* under the control of a strong promoter, such as that of *gpdA* (glyceraldehyde3-phosphate dehydrogenase) gene, produced a marked impact on the transcription of multiple SM biosynthetic genes, including those responsible for the production of sterigmatocystin, terrequinone, nidulanin A, cichorine, and emodin (Grau et al., 2019). This finding suggests that other *LaeA*-like methyltransferases may also have latent regulatory potential that becomes apparent only under conditions of derepression or overexpression.

## 14 Conclusions and future outlook

There are numerous examples, still poorly characterized, demonstrating that LaeA regulates cellular differentiation and the production of hundreds of diverse SMs across many classes of filamentous fungi, including both Ascomycetes and Basidiomycetes (Table 1). The velvet proteins are widely conserved in these groups but are absent from Hemiascomycete yeasts (Ascomycetes that form ascospores but lack fruiting bodies), such as members of the Saccharomycotina class. The extended presence of the *laeA* gene in different classes of fungi and its absence in the Saccharomycotina class raises the question of whether these genes have first evolved in an ancient progenitor common to all fungi and then has been lost in some yeasts. The loss of LaeA in Saccharomycotina class may be due to the adaptation of these yeasts to sugar-rich habitats. These yeasts show a small genome size as compared with filamentous fungi and do not contain SM gene clusters. Therefore, LaeA is not strictly required in those yeasts since they have a simple metabolism. The widespreaded presence of LaeA in most fungi correlates with its multiple effects on growth, sexual and asexual differentiation and in regulation of natural products biosynthesis. LaeA controls also secretion of excessive amounts of organic acids and of extracellular enzymes which may be dispensable, e.g., citric acid or lignocellulolytic enzymes. Regarding the control by LaeA of extracellular enzymes in some fungi, the production of these enzymes confers an ecological advantage as compared to fungi that do not produce them. However, this advantage may be dispensable in fungi since mutants are still able to grow in absence of cellulolytic enzymes on simple sugar or organic acids as sole carbon source. Another poorly known field is the possible role of LaeA on the evolutive adaptation of the extremophile fungi e.g., *Myceliophthora thermophila* (Zhao et al., 2023), that need to grow under hard environmental or nutritional conditions. This field needs further research using advanced molecular tools that are now available for some extremophiles.

**Table 1:** Organization of different components of the velvet complex in different filamentous fungi.

Fungus	Velvet complex characteristics^1^	Secondary metabolite	Reference
I. Secondary metabolites			
*Aspergillus nidulans*	LaeA, VeA, VelB, VelC, VosA	Penicillin, orsellinic acid sterigmatocystin, errequinone,	Bok and Keller (2004), Bouhired et al. (2007), and Bok et al. (2013)
*Aspergillus flavus*, *Aspergillus parasiticus*	LaeA, VeA, VelB, VelC, VosA LaeA, VeA, VelB, VelC, VosA	Aflatoxin, cyclopiazonic acid, aflatrem	Amaike and Keller (2009) and Kale et al. (2008)
*Aspergillus carbonarius*, *Aspergillus niger* *Aspergillus ochraceus*	LaeA, VeA, VelB, VelC, VosA LaeA, VeA, VelB, VelC, VosA LaeA, VeA, VelB, VelC, VosA	Ochratoxin A	Crespo-Sempere et al. (2013), Zhang M. et al. (2022), and Wang et al. (2019)
*Penicillium chrysogenum* *Acremonium chrysogenum*	LaeA, VelA, VelB, VelC, VosA LaeA, VeA, VelB, VelC, VosA	Penicillin, PR-toxin Cephalosporin C	Kosalková et al. (2009), Kopke et al. (2013), Martín (2016), Veiga et al. (2012), and Dreyer et al. (2007)
*Aspergillus terreus* *Penicillium citrinum* *Monascus pilosus*	LaeA, VeA, VelB, VelC, VosA LaeA, VelA, VelB, VelC, VosA LaeA, VeA, VeB, VeC, VosA	Lovastatin Mevastatin Compactin	Bok and Keller (2004), Xing et al. (2010), and Zhang and Miyake (2009)
*Aspergillus fumigatus*	LaeA, VeA, VelB, VelC, VosA	Siderophores, gliotoxin	Dagenais et al. (2010) and Sugui et al. (2007)
*Monascus pilosus*	LaeA, VeA, VeB, VeC, VosA	Azaphylones	Lee et al. (2013)
*Fusarium fujikuroi*	LaeA1, Vel1, Vel2, VelC, VosA	Gibberelins, fumonisin, fusarin, bikaverin, aurofusarin	Wiemann et al. (2010) and Brown et al. (2012a, 2012b)
*Fusarium verticilloides*	Lae1, Ve1, Vel2, VelC, VosA	Fumonisin, bikaverin, fusaric acid, fusarin	Butchko et al. (2012)
*Trichoderma longibranchiatum*	LaeA1, Ve1, Vel2, VelC, VosA	peptaibols	Shi et al. (2020)
*Monascus ruber*	LaeA, VeA, VelB, VelC, VosA	Azaphilones	Liu et al. (2016)
*Ganoderma lingzhi*	LaeA, VeA, VelB, VelC, VosA	Ganoderic acid	Luo et al. (2022)
*Coprinopsis cinerea*	LaeA, VeA, VelB, VelC, VosA	Coprinoferrin	Tsunematsu et al. (2019)
II. Enzymes			
*Trichoderma reesei*	LaeA, VelA, VelB, VelC, VosA	Cellulases	Aghcheh et al. (2013b) and Aghcheh et al. (2014)
*Aspergillus carbonarius*	LaeA, VeA, VelB, VelC, VosA	Endoglucanases	Linde et al. (2016)
*Mycelliophthora thermophila*	LaeA, VeA, VelB, VelC, VosA	Celullolytic enzymes	Zhao et al. (2023)
III. Organic acids			
*Aspergillus oryzae* *Aspergillus luchuensis*	LaeA, VeA, VelB, VelC, VosA LaeA, VeA, VelB, VelC, VosA	Kojic acid	Oda et al. (2011)
*Aspergillus fumisynnematus*	LaeA, VeA, VelB, VelC, VosA	Cyclopiazonic acid	Hong et al. (2015)
*Aspergillus niger*	LaeA, VeA, VelB, VelC, VosA	Citric acid	Niu et al. (2015)
*Aspergillus carbonarius*	LaeA, VeA, VelB, VelC, VosA	Citric acid	Linde et al. (2016)
IV. Pathogenicity and virulence			
*Aspergillus fumigatus*	LaeA, VeA, VelB, VelC, VosA	Pathogenicity	Sugui et al. (2007) and Dhingra et al. (2013)
*Aspergillus flavus*	LaeA, VeA, VelB, VelC, VosA	Pathogenicity	Amaike and Keller (2009) and Chang et al. (2012)
*Botrytis cinerea*	LaeA1, Ve1, Ve2, VelC, VosA	Differentiation/oxalic acid/Pathogenicity	Yang et al. (2013) and Schumacher et al. (2015)
*Fusarium oxysporum*	LaeA1, VelA, VelB, VelC, VosA	Beauvericin/Pathogenicity	López-Berges et al. (2013)
*Fusarium graminearum*	LaeA1, Vel1, Vel2, VelC, VosA	Virulence/ Pathogenicity	Jiang et al. (2011) and Kim et al. (2013)
*Cochliobolus heterostrophus*	LaeA, VelA, VelB, VelC, VosA	T-toxin, melanin/virulence	Wu et al. (2012)
*Alternaria alternata*	LaeA, VeA, VelB, VelC, VosA	Toxins/ pathogenicity	Takao et al. (2016) and Wang et al. (2022)

Indicated in blue are those proteins in which some experimental research has been done. Proteins indicated in green have well bioinformatically characterized genes, but no experimental work has been made with them. The proteins labeled in red have no homologous proteins to *A. nidulans* (as it is in the case of *Ganoderma* and *Monascus* sp.) or have only similar domains but very low similarity. These proteins may not be present in the fungus genome and should be further studied.

The velvet proteins VeA, VelB, VelC, LaeA, and VosA, are present in all *Aspergillus* and *Penicilium* species. Bioinformatic analysis indicates that LaeA is present in *Ganoderma* and *Monascus* species. In fact, the absence of the *vosA* gene in *Fusarium* species and of *velC* and *vosA* in *A. chrysogenum* has been reported previously (López-Berges et al., 2013; Terfehr et al., 2017). The best conserved component is VelB while VelC and VosA show a great variability (Table 1), therefore these proteins should be studied in depth to conclude their presence in different fungi (Chen et al., 2024).

In *A. nidulans*, *A. parasiticus* and *A. flavus*, it is well established that the velvet complex exerts its regulatory function through the interaction of VeA, VelB, and LaeA, where VeA acts as a scaffold protein facilitating the association between VelB and LaeA (Bayram et al., 2008). However, in some species of the *Fusarium* genus, the interactions among velvet proteins differ from the *Aspergillus* model. For instance, in *F. graminearum*, the VeA protein (FgVeA) has been well characterized, yet evidence indicates that it does not form a close interaction with either VelB or LaeA (Jiang et al., 2011).

Importantly, the effect of LaeA on SM biosynthesis is not necessarily dependent on its interaction with VeA. Mutants of *F. fujikuroi* and *P. chrysogenum* VelA-defective still exhibit LaeA dependent significant modification in SM production, indicating that LaeA can regulate these pathways independently of VeA in such strains (Wiemann et al., 2010; Hoff et al., 2010). These findings suggest that, although cooperative interactions between velvet-proteins play a major role in transcriptional regulation of multiple genes (Bayram et al., 2008, 2012), the individual components in some fungi also exert regulatory functions on their own.

The regulatory activity of LaeA is exerted at least at two distinct levels: (1) binding of LaeA to specific DNA sequences, and (2) possible methylation of substrate proteins. First, VosA of *A. nidulans,* VelA of *P. chrysogenum* and the Rye3 (homologous to VelB) and Rye2 of *H. capsulatum*—recognize specific 11-nucleotide sequences within the promoter of target genes, thereby activating or repressing their transcription (Figure 4) (Ahmed et al., 2013; Beyhan et al., 2013; Becker et al., 2016). These authors identified the velvet-motif sequence and demonstrated the binding *in vitro* using electrophoretic mobility shift assays. They observed that Ryp3 and Ryp2 bind to target-gene promoters (Figure 4) is enhanced when both proteins are co-expressed, as shown in heterologous assays performed in *Saccharomyces cerevisiae*. Recently, LaeA and VeA binding Response Elements in *A. nidulans* have been reported (Figure 4; Moon et al., 2023).

Notably, interdependent regulation among components of the velvet system has been documented in several fungi. For example, VeA regulates expression of *laeA* in *A. nidulans*, *P. chrysogenum* and *F. fujikuroi* (Amaike and Keller, 2009; Hoff et al., 2010; Wiemann et al., 2010) and, conversely, LaeA is required for expression of *veA* in *T. reesei* (Aghcheh et al., 2013b) while LaeA represses VeA in *A. flavus* (Kale et al., 2008). The velvet components Ryp2 and Ryp3 in *H. capsulatum* were observed to interact with non-velvet proteins such as Ryp1, a regulatory protein controlling acetate utilization, in coordinating the expression of target genes (Beyhan et al., 2013). Altogether, these findings support the increasingly evident notion that velvet components and other regulatory protein complexes cooperate extensively to control the expression of genes involved in secondary metabolism and cellular differentiation. Indeed, *laeA* expression itself is regulated by several transcription factors, including the binuclear Zn (II)₂Cys₆ regulator AflR in *A. nidulans* (Bok and Keller, 2004), and the MclR transcriptional regulator in the mevastatin producer *P. citrinum* (Xing et al., 2010). Recently, interactions between velvet-complex components and other transcription factors in *A. nidulans* have been reported (Moon et al., 2023). The use of advanced molecular omic tools and protein chemistry would be helpful to clarify the molecular mechanisms underlying these interactions and their impact on secondary metabolism and cellular differentiation.

Increasing evidence indicates that LaeA, together with other velvet proteins, also regulates transporter genes responsible for secreting SMs and other extracellular signaling molecules that mediate inter-organism communication. Particularly noteworthy is the regulation of siderophore biosynthesis in the Basidiomycetes. *C. cinerea* (Tsunematsu et al., 2019) and in certain ascomycetes, such as *A. fumigatus* (Perrin et al., 2007). However, information on the LaeA regulation of siderophores biosynthesis in other ascomycetes remains fragmented.

Much remains to be discovered about how LaeA and the velvet complex influence the secretion of metabolites, many of which are exported through vesicle-mediated pathways (Martín and Liras, 2024), e.g., the *A. chrysogenum* cephalosporin gene cluster contains three MFS transporters. Mutants defective in two of these genes (*cefM* and *cefP*) block cephalosporin biosynthesis and secretion (Teijeira et al., 2009; Ullan et al., 2010). In this context, the LaeA-dependent control of citric acid secretion, mediated by the CexA transporter protein, represents a novel important example of LaeA role in regulating secretion of small molecules via major facilitator superfamily transporters.

Second, the LaeA global regulator contains a conserved binding domain characteristic of methyltransferases, and there is substantial evidence that this domain is essential for LaeA function. For this reason, LaeA has been proposed to act as a putative methyltransferase, although its physiological substrate remains unknown despite efforts by several research groups to identify the target molecule.

The LaeA putative methyltransferase activity has been suggested in some cases to be involved in methylation of lysine residues on histones H3 and H4. Several histone methyltransferases are known to modify histones H3 and H4; these enzymes are likely distinct from LaeA (Freitag, 2017). Low expression of SM biosynthetic genes is typically associated with heterochromatin markers, including trimethylated lysine histones and with hight level of heterochromatin associated protein A (HPA) (Reyes-Domínguez et al., 2010; Strauss and Reyes-Domínguez, 2011). Activation of these biosynthetic clusters requires chromatin remodeling, specifically a reduction in H3K9 trimethylation and a decrease in HPA level (Reyes-Domínguez et al., 2010; Strauss and Reyes-Domínguez, 2011). These authors proposed that LaeA counteracts formation of the heterochromatin structure through protein interactions with histones, thereby promoting a shift toward euchromatin that facilitates gene expression. Furthermore, it has been unequivocally established that the expression levels of SM biosynthetic genes are strongly influenced by diamines and triamines that modify the degree of condensation of histones thereby affecting heterochromatin structure (Martín, 2025). Additional work is needed to elucidate the mechanism linking the putative LaeA methyltransferase activity with the recognition of specific DNA sequences that positively or negatively influence the expression of target genes; this remains an open question. This research will require utilization of protein chemistry, including expression in yeasts to confirm *in vitro* the DNA-protein interaction by electrophoretic mobility shift assays.

## References

[ref1] Aghcheh R. K. Bok J. W. Phatale P. A. Smith K. M. Baker S. E. Lichius A. . (2013b). Functional analyses of *Trichoderma reesei* LAE1 reveal conserved and contrasting roles of this regulator. G3 (Bethesda) 3, 369–378. doi: 10.1534/g3.112.005140, 23390613 PMC3564997

[ref2] Aghcheh R. K. Druzhinina I. S. Kubicek C. P. (2013a). The putative protein methyltransferase LAE1 of *Trichoderma atroviride* is a key regulator of asexual development and mycoparasitism. PLoS One 8:e67144. doi: 10.1371/journal.pone.0067144, 23826217 PMC3691206

[ref3] Aghcheh R. K. Németh Z. Atanasova L. Fekete E. Paholcsek M. Sándor E. . (2014). The VELVET an orthologue VEL1 of *Trichoderma reesei* regulates fungal development and is essential for cellulase gene expression. PLoS One 9:e112799. doi: 10.1371/journal.pone.0112799, 25386652 PMC4227869

[ref4] Ahmad M. F. Wahab S. Ahmad F. A. Ashraf S. A. Abullais S. S. Saas H. H. (2022). *Ganoderma lucidum*, a potential pleiotropic approach of ganoderic acids in health reinforcement and factors influencing their production. Fungal Biol. Rev. 39, 100–125. doi: 10.1016/j.fbr.2021.12.003

[ref5] Ahmed Y. L. Gerke J. Park H. S. Bayram O. Neumann P. Ni M. . (2013). The velvet family of fungal regulators contains a DNA-binding domain structurally similar to NF-kB. PLoSBiol. 11:e1001750. doi: 10.1371/journal.pbio.1001750, 24391470 PMC3876986

[ref6] Akagi Y. Taga M. Yamamoto M. Tsuge T. Fukumasa-Nakai Y. Otani H. . (2009). Chromosome constitution of hybrid strains constructed by protoplast fusion between the tomato and strawberry pathotypes of *Alternaria alternata*. J. Gen. Plant Pathol. 75, 101–109. doi: 10.1007/s10327-009-0149-1

[ref7] Amaike S. Keller N. P. (2009). Distinct roles for VeA and LaeA in development and pathogenesis of *Aspergillus flavus*. Eukaryot. Cell 8, 1051–1060. doi: 10.1128/EC.00088-09, 19411623 PMC2708460

[ref8] Atoui A. Bao D. Kaur N. Grayburn N. W. Calvo A. M. (2008). *Aspergillus nidulans* natural product biosynthesis is regulated by MpkB, a putative pheromone response mitogen-activated protein kinase. Appl. Env. Microbiol. 74, 3596–3600. doi: 10.1128/AEM.02842-07, 18378656 PMC2423048

[ref9] Bardwell L. (2005). A walk-through of the yeast mating pheromone response pathway. Peptides 26, 339–350. doi: 10.1016/j.peptides.2004.10.002, 15690603 PMC3017506

[ref10] Bartoshevich Y. E. Zaslavskaya P. L. Novak M. J. Yudina O. D. (1990). Acremonium chrysogenum differentiation and biosynthesis of cephalosporin. J. Basic Microbiol. 30, 313–320. doi: 10.1002/jobm.3620300503, 2213533

[ref11] Bayram O. Bayram O. S. Ahmed Y. L. Maruyama J. I. Valerius O. Rizzoli S. O. . (2012). The *Aspergillus nidulans* MAPK module AnSte11-Ste50-Ste7-Fus3 controls development and secondary metabolism. PLoS Genet. 8:e1002816. doi: 10.1371/journal.pgen.1002816, 22829779 PMC3400554

[ref12] Bayram O. Braus G. H. (2012). Coordination of secondary metabolism and development in fungi: the velvet family of regulatory proteins. FEMS Microbiol. Rev. 36, 1–24. doi: 10.1111/j.1574-6976.2011.00285.x, 21658084

[ref13] Bayram O. Krappmann S. Ni M. Bok J. Helmstaedt K. Valerius O. . (2008). VelB/VeA/LaeA complex coordinates light signal with fungal development and secondary metabolism. Science 320, 1504–1506. doi: 10.1126/science.1155888, 18556559

[ref14] Bayram S. O. Palmer J. M. Keller N. P. Braus G. H. Bayram O. (2015). One Juliet and four Romeos: VeA and its methyltransferases. Front. Microbiol. 6:1. doi: 10.3389/fmicb.2015.00001, 25653648 PMC4299510

[ref15] Bayram O. Sari F. Braus G. H. Irniger S. (2009). The protein kinase ImeB is required for light-mediated inhibition of sexual development and for mycotoxin production in *Aspergillus nidulans*. Mol. Microbiol. 71, 1278–1295. doi: 10.1111/j.1365-2958.2009.06606.x, 19210625

[ref16] Becker K. Ziemons S. Lentz K. Freitag M. Kück U. (2016). Genome-wide chromatin immunoprecipitation sequencing analysis of the *Penicillium chrysogenum* velvet protein PcVelA identifies methyltransferase PcLlmA as a novel downstream regulator of fungal development. mSphere 1:e00149. doi: 10.1128/mSphere.00149-16, 27570838 PMC4999599

[ref17] Beyhan S. Gutiérrez M. Voorhies M. Sil A. (2013). A temperature-responsive network links cell shape and virulence traits in a primary fungal pathogen. PLoS Biol. 11:e1001614. doi: 10.1371/journal.pbio.1001614, 23935449 PMC3720256

[ref18] Bi Q. Wu D. Zhu X. Turgeon B. G. (2013). *Cochliobolus heterostrophus* Llm1, a Lae1-like methyltransferase regulates T-toxin production, virulence and development. Fungal Genet. Biol. 51, 21–33. doi: 10.1016/j.fgb.2012.11.012, 23261970

[ref19] Blum M. Andreeva A. Florentino L. C. Chuguransky S. R. Grego T. Hobbs E. . (2025). InterPro: the protein sequence classification resource in 2025. Nucleic Acids Res. 53, D444–D456. doi: 10.1093/nar/gkae1082, 39565202 PMC11701551

[ref20] Bok J. W. Keller N. P. (2004). LaeA, a regulator of secondary metabolism in *Aspergillus* spp. Eukaryot. Cell 3, 527–535. doi: 10.1128/EC.3.2.527-535.2004, 15075281 PMC387652

[ref21] Bok J. W. Soukup A. A. Chadwick E. Chiang Y. M. Wang C. C. Keller N. P. (2013). VeA and MvlA repression of the cryptic orsellinic acid gene cluster in *Aspergillus nidulans* involves histone 3 acetylation. Mol. Microbiol. 89, 963–974. doi: 10.1111/mmi.12326, 23841751 PMC3773851

[ref22] Bouhired S. Weber M. Kempf-Sontag A. Keller N. P. Hoffmeister D. (2007). Accurate prediction of the *Aspergillus nidulans* terrequinone gene cluster boundaries using the transcriptional regulator LaeA. Fungal Genet. Biol. 44, 1134–1145. doi: 10.1016/j.fgb.2006.12.010, 17291795

[ref23] Brandenburger E. Gressler M. Leonhardt R. Lackner G. Habel A. Hertweck C. . (2017). A highly conserved basidiomycete peptide Synthetase produces a trimeric Hydroxamate siderophore. Appl. Environ. Microbiol. 83, e01478–e01417. doi: 10.1128/AEM.01478-17, 28842536 PMC5648918

[ref24] Brown D. W. Butchko R. A. E. Baker S. E. Proctor R. H. (2012a). Phylogenomic and functional domain analysis of polyketide synthases in fusarium. Fungal Biol. 116, 318–331. doi: 10.1016/j.funbio.2011.12.005, 22289777

[ref25] Brown D. W. Butchko R. A. E. Busman M. Proctor R. H. (2012b). Identification of gene clusters associated with fusaric acid, fusarin, and perithecial pigment production in *Fusarium verticillioides*. Fungal Genet. Biol. 49, 521–532. doi: 10.1016/j.fgb.2012.05.010, 22652150

[ref26] Bushley K. E. Ripoll D. R. Turgeon B. G. (2008). Module evolution and substrate specificity of fungal nonribosomal peptide synthetases involved in siderophore biosynthesis. BMC Evol. Biol. 8:328. doi: 10.1186/1471-2148-8-328, 19055762 PMC2644324

[ref27] Butchko R. A. Brown D. W. Busman M. Tudzynski B. Wiemann P. (2012). Lae1 regulates expression of multiple secondary metabolite gene clusters in *Fusarium verticillioides*. Fungal Genet. Biol. 49, 602–612. doi: 10.1016/j.fgb.2012.06.003, 22713715

[ref28] Calvo A. M. Bok J. Brooks W. Keller N. P. (2004). veA is required for toxin and sclerotial production in *Aspergillus parasiticus*. Appl. Environ. Microbiol. 70, 4733–4739. doi: 10.1128/AEM.70.8.4733-4739.2004, 15294809 PMC492383

[ref29] Campoy S. Rumbero A. Martín J. F. Liras P. (2005). Characterization of an hyperpigmenting mutant of *Monascus purpureus* IB1: identification of two novel pigment chemical structures. Appl. Microbiol. Biotechnol. 70, 488–496. doi: 10.1007/s00253-005-0090-y, 16151799

[ref30] Castrillo M. Luque E. M. Pardo-Medina J. Limon M. C. Corrochano L. M. Avalos J. (2018). Transcriptional basis of enhanced photoinduction of carotenoid biosynthesis at low temperature in the fungus *Neurospora crassa*. Res. Microbiol. 169, 78–89. doi: 10.1016/j.resmic.2017.11.003, 29203212

[ref31] Chang P. K. Scharfenstein L. L. Ehrlich K. C. Wei Q. Bhatnagar D. Ingber B. F. (2012). Effects of laeA deletion on *Aspergillus flavus* conidial development and hydrophobicity may contribute to loss of aflatoxin production. Fungal Biol. 116, 298–307. doi: 10.1016/j.funbio.2011.12.003, 22289775

[ref32] Chen W., Son Y. E. Cho H. J., Choi D. Park H. S. Yu J. H. (2024). Phylogenomics analysis of velvet regulators in the fungal kingdom. Microbiol. Spectr. 212:e071723. doi: 10.1128/spectrum.03717-23 PMC10845976 38179919

[ref33] Chen N. H. Liu J. W. Zhong J. J. (2008). Ganoderic acid inhibits tumor invasion through downregulating matrix metalloproteinases 2/9 gene expression. J. Pharmacol. Sci. 108, 212–216. doi: 10.1254/jphs.sc0080019, 18946196

[ref34] Chen S. L. Xu J. Liu C. Zhu Y. J. Nelson D. R. Zhou S. G. . (2012). Genome sequence of the model medicinal mushroom *Ganoderma lucidum*. Nat. Commun. 3:913. doi: 10.1038/ncomms1923 22735441 PMC3621433

[ref35] Crespo-Sempere A. Marín S. Sanchis V. Ramos A. J. (2013). VeA and LaeA transcriptional factors regulate ochratoxin A biosynthesis in *Aspergillus carbonarius*. Int. J. Food Microbiol. 166, 479–486. doi: 10.1016/j.ijfoodmicro.2013.07.027, 24041999

[ref36] Dagenais T. R. Giles S. S. Aimanianda V. Latge J. P. Hull C. M. Keller N. P. (2010). *Aspergillus fumigatus* LaeA mediated phagocytosis is associated with a decreased hydrophobin layer. Infect. Immun. 78, 823–829. doi: 10.1128/IAI.00980-09, 19917717 PMC2812189

[ref37] Dean R. Van Kan J. A. Pretorius Z. A. Hammond-Kosack K. E. Di Pietro A. Spanu P. D. . (2012). The top 10 fungal pathogens in molecular plant pathology. Mol. Plant Pathol. 13, 414–430. doi: 10.1111/j.1364-3703.2011.00783.x, 22471698 PMC6638784

[ref38] Dhingra S. Lind A. L. Lin H. C. Tang Y. Rokas A. Calvo A. M. (2013). The fumagillin gene cluster, an example of hundreds of genes under veA control in *Aspergillus fumigatus*. PLoS One 8:e77147. doi: 10.1371/journal.pone.0077147, 24116213 PMC3792039

[ref39] Dreyer J. Eichhorn H. Friedlin E. Kürnsteiner H. Kück U. (2007). A homologue of the *Aspergillus* velvet gene regulates both cephalosporin C biosynthesis and hyphal fragmentation in *Acremonium chrysogenum*. Appl. Environ. Microbiol. 73, 3412–3422. doi: 10.1128/AEM.00129-07, 17400783 PMC1907097

[ref40] Duran R. M. Cary J. W. Calvo A. M. (2007). Production of cyclopiazonic acid, aflatrem, and aflatoxin by *Aspergillus flavus* is regulated by veA, a gene necessary for sclerotial formation. Appl. Microbiol. Biot. 73, 1158–1168. doi: 10.1007/s00253-006-0581-5, 16988822

[ref41] Ehrlich K. C. Yu J. Cotty P. J. (2005). Aflatoxin biosynthesis gene clusters and flanking regions. J. Appl. Microbiol. 99, 518–527. doi: 10.1111/j.1365-2672.2005.02637.x, 16108793

[ref42] Erdős G. Dosztányi Z. (2020). Analyzing protein disorder with IUPred2A. Curr. Protoc. Bioinformatics 70:e99. doi: 10.1002/cpbi.99, 32237272

[ref43] Ferrara M. Gallo A. Cervini C. Gambacorta L. Solfrizzo M. Baker S. E. . (2021). Evidence of the involvement of a cyclase gene in the biosynthesis of ochratoxin a in *Aspergillus carbonarius*. Toxins (Basel) 13:892. doi: 10.3390/toxins13120892, 34941729 PMC8705981

[ref44] Fierro F. Barredo J. L. Díez B. Gutiérrez S. Fernández F. J. Martín J. F. (1995). The penicillin gene cluster is amplified in tandem repeats linked by conserved hexanucleotide sequences. Proc. Natl. Acad. Sci. U. S. A. 92, 6200–6204. doi: 10.1073/pnas.92.13.6200, 7597101 PMC41670

[ref45] Finn R. D. Coggill P. Eberhardt R. Y. Eddy S. R. Mistry J. Alex L. . (2016). The Pfam protein families database: towards a more sustainable future. Nucleic Acids Res. 44, D279–D285. doi: 10.1093/nar/gkv1344, 26673716 PMC4702930

[ref46] Fischer M. S. Glass N. L. (2019). Communicate and fuse: how filamentous fungi establish and maintain an interconnected mycelial network. Front. Microbiol. 10:619. doi: 10.3389/fmicb.2019.00619, 31001214 PMC6455062

[ref47] Fontecave M. Atta M. Mulliez E. (2004). S-adenosyl methionine: nothing goes to waste. Trends Biochem. Sci. 29, 243–249. doi: 10.1016/j.tibs.2004.03.007, 15130560

[ref48] Frawley D. Stroe M. C. Oakley B. R. Heinekamp T. Straßburger M. Fleming A. B. . (2020). The pheromone module SteC-MkkB-MpkB-SteD-HamE regulates development, stress responses and secondary metabolism in *Aspergillus fumigatus*. Frontiers Microbiol. 11:811. doi: 10.3389/fmicb.2020.00811, 32457716 PMC7223695

[ref49] Freitag M. (2017). Histone methylation by SET domain proteins in Fungi. Ann. Rev. Microbiol. 71, 413–439. doi: 10.1146/annurev-micro-102215-095757, 28715960

[ref50] García-Estrada C. Barreiro C. Martín J. F. (2025). Potential and representative anticancer secondary metabolites produced by fungi. Int. J. Mol. Sci. 27:101. doi: 10.3390/ijms27010101, 41515980 PMC12785832

[ref51] García-Estrada C. Ullán R. V. Albillos S. M. Fernández-Bodega M. A. Durek P. Von Döhren H. . (2011). A single cluster of coregulated genes encodes the biosynthesis of the mycotoxins roquefortine C and meleagrin in *Penicillium chrysogenum*. Chem. Biol. 18, 1499–1512. doi: 10.1016/j.chembiol.2011.08.012, 22118684

[ref52] García-Rico R. Fierro F. Martín J. F. (2008). Heterotrimeric gα protein Pga1 of *Penicillium chrysogenum* controls conidiation mainly by a cAMP-independent mechanism and its deletion leads to a sporulation microcycle in liquid cultures. Biochem. Cell Biol. 86, 57–69. doi: 10.1139/o07-148, 18364746

[ref53] Godio R. P. Fouces R. Martín J. F. (2007). A squalene epoxidase is involved in biosynthesis of both the antitumor compound Clavaric acid and sterols in the basidiomycete *H. sublateritium*. Chem. Biol. 14, 1334–1346. doi: 10.1016/j.chembiol.2007.10.018, 18096502

[ref54] Godio R. P. Martín J. F. (2009). Modified oxidosqualene cyclases in the formation of bioactive secondary metabolites: biosynthesis of the antitumor clavaric acid. Fungal Genet. Biol. 46, 232–242. doi: 10.1016/j.fgb.2008.12.002, 19130892

[ref55] Grau M. F. Entwistle R. Wang C. C. C. Oakley B. R. (2019). Overexpression of an LaeA-like methyltransferase upregulates secondary metabolite production in *Aspergillus nidulans*. ACS Chem. Biol. 14, 1643–1651. doi: 10.1021/acschembio.9b00380, 31265232 PMC7310610

[ref57] Gutiérrez S. Fierro F. Casqueiro J. Martin J. F. (1999). Gene organization and plasticity of the b-lactam genes in different filamentous fungi. Ant. Van Leeuvenhoek 75, 81–94. doi: 10.1023/a:1001861025070, 10422582

[ref58] Hai Y. Jenner M. Tang Y. (2020). Fungal siderophore biosynthesis catalysed by an iterative nonribosomal peptide synthetase. Chem. Sci. 11, 11525–11530. doi: 10.1039/d0sc03627g, 34094397 PMC8162485

[ref59] Harimoto Y. Hatta R. Kodama M. Yamamoto M. Otani H. Tsuge T. (2007). Expression profiles of genes encoded by the supernumerary chromosome controlling AM-toxin biosynthesis and pathogenicity in the apple pathotype of *Alternaria alternata*. Mol. Plant-Microbe Interact. 20, 1463–1476. doi: 10.1094/MPMI-20-12-1463, 17990954

[ref60] Hidalgo P. I. Ullán R. V. Albillos S. M. Montero O. Fernández-Bodega M. Á. García-Estrada C. . (2014). Molecular characterization of the PR-toxin gene cluster in *Penicillium roqueforti* and *Penicillium chrysogenum*: cross talk of secondary metabolite pathways. Fungal Genet. Biol. 62, 11–24. doi: 10.1016/j.fgb.2013.10.009, 24239699

[ref61] Hoff B. Kamerewerd J. Sigl C. Mitterbauer R. Zadra I. Kürnsteiner H. . (2010). Two components of a velvet-like complex control hyphal morphogenesis, conidiophore development, and penicillin biosynthesis in *Penicillium chrysogenum*. Eukaryot. Cell 9, 1236–1250. doi: 10.1128/EC.00077-10, 20543063 PMC2918941

[ref62] Hong E. J. Kim N. K. Lee D. Kim W. G. Lee I. (2015). Overexpression of the laeA gene leads to increased production of cyclopiazonic acid in *Aspergillus fumisynnematus*. Fungal Biol. 119, 973–983. doi: 10.1016/j.funbio.2015.06.006, 26466873

[ref63] Hou Z. Xue C. Peng Y. Katan T. Kistler H. C. Xu J. R. (2002). A mitogen-activated protein kinase gene (MGV1) in *Fusarium graminearum* is required for female fertility, heterokaryon formation, and plant infection. Mol. Plant-Microbe Interact. 15, 1119–1127. doi: 10.1094/MPMI.2002.15.11.1119, 12423017

[ref64] Imazaki A. Tanaka A. Harimoto Y. Yamamoto M. Akimitsu K. Park P. . (2010). Contribution of peroxisomes to secondary metabolism and pathogenicity in the fungal plant pathogen *Alternaria alternata*. Eukaryot. Cell 9, 682–694. doi: 10.1128/EC.00369-09, 20348386 PMC2863954

[ref65] Jiang J. Liu X. Yin Y. Ma Z. (2011). Involvement of a velvet protein FgVeA in the regulation of asexual development, lipid and secondary metabolisms and virulence in *Fusarium graminearum*. PLoS One 6:e28291. doi: 10.1371/journal.pone.0028291, 22140571 PMC3226687

[ref66] Jun S. C. Kim J. H. Han K. H. (2020). The conserved MAP kinase MpkB regulates development and sporulation without affecting aflatoxin biosynthesis in *Aspergillus flavus*. J. Fungi 6:289. doi: 10.3390/jof6040289, 33207581 PMC7711526

[ref67] Kadooka C. Nakamura E. Mori K. Okutsu K. Yoshizaki Y. Takamine K. . (2020). LaeA controls citric acid production through regulation of the citrate exporter encoding cexA gene in *Aspergillus luchuensis* Mut. Kawachii. Appl. Environ. Microbiol. 86:e01950. doi: 10.1128/AEM.01950-19, 31862728 PMC7028977

[ref68] Kale S. P. Milde L. Trapp M. K. Frisvad J. C. Keller N. P. Bok J. W. (2008). Requirement of LaeA for secondary metabolism and sclerotial production in *Aspergillus flavus*. Fungal Genet. Biol. 45, 1422–1429. doi: 10.1016/j.fgb.2008.06.009, 18667168 PMC2845523

[ref69] Kang Y. Jeesun C. J. Jun S.-C. Han D.-M. Chae K.-S. Jahng K. Y. (2013). The MpkB MAP kinase plays a role in autolysis and conidiation of *Aspergillus nidulans*. Fungal Gen. Biol. 61, 42–49. doi: 10.1016/j.fgb.2013.09.010, 24161728

[ref70] Kim H. K. Lee S. Jo S. M. McCormick S. P. Butchko R. A. Proctor R. H. . (2013). Functional roles of FgLaeA in controlling secondary metabolism, sexual development, and virulence in *Fusarium graminearum*. PLoS One 8:e68441. doi: 10.1371/journal.pone.0068441, 23874628 PMC3713025

[ref71] Kopke K. Hoff B. Bloemendal S. Katschorowski A. Kamerewerd J. Kück U. (2013). Members of the velvet complex play functionally opposing roles in the regulation of penicillin biosynthesis and conidiation. Eukaryotic Cells 12, 299–310. doi: 10.1128/EC.00272-12, 23264641 PMC3571298

[ref72] Kosalková K. García-Estrada C. Ullán R. V. Godio R. P. Feltrer R. Teijeira F. . (2009). The global regulator LaeA controls penicillin biosynthesis, pigmentation and sporulation, but not roquefortine C synthesis in *Penicillium chrysogenum*. Biochimie 91, 214–225. doi: 10.1016/j.biochi.2008.09.004, 18952140

[ref73] Kubicek C. P. Steindorff A. S. Chenthamara K. Manganiello G. Henrissat B. Zhang J. . (2019). Evolution and comparative genomics of the most common *Trichoderma* species. BMC Genomics 20:485. doi: 10.1186/s12864-019-5680-7, 31189469 PMC6560777

[ref74] Lai Y. Wang L. Zheng W. Wang S. (2022). Regulatory roles of histone modifications in filamentous fungal pathogens. J. Fungi 8:565. doi: 10.3390/jof8060565, 35736048 PMC9224773

[ref75] Lee S. S. Lee J. H. Lee I. (2013). Strain improvement by overexpression of the laeA gene in Monascus pilosus for the production of monascus-fermented rice. J. Microbiol. Biotechnol. 23, 959–965. doi: 10.4014/jmb.1303.03026, 23727802

[ref76] Li J. Lin L. Sun T. Xu J. Ji J. Liu Q. . (2020). Direct production of commodity chemicals from lignocellulose using *Myceliophthora thermophila*. Metabol. Eng. 61, 416–426. doi: 10.1016/j.ymben.2019.05.007, 31078793

[ref77] Li J. Liu G. Chen M. Li Z. Quin Y. Qu Y. (2013). Cellodextrin transporters play important roles in cellulase induction in the cellulolytic fungus *Penicillium oxalicum*. Appl. Microbiol. Biotechnol. 97, 10479–10488. doi: 10.1007/s00253-013-5301-3, 24132667

[ref78] Li Z. Yao G. Wu R. Gao L. Kan Q. Liu M. . (2015). Synergistic and dose-controlled regulation of cellulase gene expression in *Penicillium oxalicum*. PLoS Genet. 11:e1005509. doi: 10.1371/journal.pgen.1005509, 26360497 PMC4567317

[ref79] Li Y. Zheng X. Zhang X. Bao L. Zhu Y. Qu Y. . (2016). The different roles of *Penicillium oxalicum* LaeA in the production of extracellular cellulase and β-xylosidase. Front. Microbiol. 7:2091. doi: 10.3389/fmicb.2016.02091, 28066400 PMC5177634

[ref80] Linde T. Zoglowek M. Lübeck M. Frisvad J. C. Lübeck P. S. (2016). The global regulator LaeA controls production of citric acid and endoglucanases in *Aspergillus carbonarius*. J. Ind. Microbiol. Biotechnol. 43, 1139–1147. doi: 10.1007/s10295-016-1781-3, 27169528

[ref81] Linz J. E. Wee J. M. Roze L. V. (2014). “Aflatoxin biosynthesis, regulation and subcellular localization” in Biosynthesis and molecular genetics of fungal secondary metabolites. eds. Martín J. F. García-Estrada C. Zeilinger S. (New York: Springer), 89–110.

[ref82] Liras P. Martín J. F. (2023). Interconnected set of enzymes provide lysine biosynthetic intermediates and ornithine derivatives as key precursors for the biosynthesis of bioactive secondary metabolites. Antibiotics (Basel) 12:159. doi: 10.3390/antibiotics12010159, 36671360 PMC9854754

[ref83] Liu Q. Cai L. Shao Y. Zhou Y. Li M. Wang X. . (2016). Inactivation of the global regulator LaeA in *Monascus ruber* results in a species-dependent response in sporulation and secondary metabolism. Fungal Biol. 120, 297–305. doi: 10.1016/j.funbio.2015.10.008, 26895858

[ref84] López-Berges M. S. Hera C. Sulyok M. Schäfer K. Capilla J. Guarro J. . (2013). The velvet complex governs mycotoxin production and virulence of *Fusarium oxysporum* on plant and mammalian hosts. Mol. Microbiol. 87, 49–65. doi: 10.1111/mmi.12082, 23106229

[ref85] Luo Q. Li N. Xu J. W. (2022). A methyltransferase LaeA regulates ganoderic acid biosynthesis in *Ganoderma lingzhi*. Front. Microbiol. 13:1025983. doi: 10.3389/fmicb.2022.1025983, 36312944 PMC9614229

[ref9001] Ma D. Li R. (2013). Current understanding of HOG-MAPK pathway in Aspergillus fumigatus. Mycopathologia. 175, 13–23., 23161019 10.1007/s11046-012-9600-5

[ref86] Marik T. Tyagi C. Balázs D. Urbán P. Szepesi Á. Bakacsy L. . (2019). Structural diversity and bioactivities of peptaibol compounds from the Longibrachiatum clade of the filamentous fungal genus *Trichoderma*. Front. Microbiol. 10:1434. doi: 10.3389/fmicb.2019.01434, 31293557 PMC6606783

[ref87] Martín J. F. (2016). Key role of LaeA and velvet complex proteins on expression of β-lactam and PR-toxin genes in *Penicillium chrysogenum*: cross-talk regulation of secondary metabolite pathways. J. Ind. Microbiol. Biotechnol. 44, 525–535. doi: 10.1007/s10295-016-1830-y, 27565675

[ref88] Martín J. F. (2025). Polyamine induction of secondary metabolite biosynthetic genes in fungi is mediated by global regulator LaeA and α-NAC transcriptional coactivator: connection to epigenetic modification of histones. Molecules 30:3903. doi: 10.3390/molecules30193903, 41097324 PMC12526404

[ref89] Martín J. F. Coton M. (2016). “Blue cheese: microbiota and fungal metabolites” in Fermented foods in health and disease prevention. eds. Frias J. Martínez-Villaluenga J. C. Peñas E. (New York: Elsevier), 275–304.

[ref90] Martín J. F. Demain A. L. (2002). Unraveling the methionine-cephalosporin puzzle in *Acremonium chrysogenum*. Trends Biotechnol. 20, 12502–12507. doi: 10.1016/s0167-7799(02)02070-x, 12443871

[ref91] Martín J. F. Liras P. (2015). “Novel antimicrobial and other bioactive metabolites obtained from silent gene clusters” in Antibiotics: Current innovations and future trends. eds. Demain A. L. Sánchez S. (Norfolk, UK: Horizon Scientific Press and Caister Academic Press), 275–292.

[ref92] Martín J. F. Liras P. (2024). Targeting of specialized metabolites biosynthetic enzymes to membranes and vesicles by posttranslational palmitoylation: a mechanism of non-conventional traffic and secretion of fungal metabolites. Int. J. Mol. Sci. 25:1224. doi: 10.3390/ijms25021224, 38279221 PMC10816013

[ref93] Martín J. F. Van den Berg M. A. Ver Loren van Themaat E. Liras P. (2019). Sensing and transduction of nutritional and chemical signals in filamentous fungi: impact on cell development and secondary metabolites biosynthesis. Biotechnol. Adv. 37:107392. doi: 10.1016/j.biotechadv.2019.04.014 31034961

[ref94] Martínez D. Berka R. M. Henrissat B. Saloheimo M. Arvas M. Baker S. E. . (2008). Genome sequencing and analysis of the biomass-degrading fungus *Trichoderma reesei* (syn. *Hypocrea jecorina*). Nat. Biotechnol. 26, 553–560. doi: 10.1038/nbt1403, 18454138

[ref95] Merhej J. Urban M. Dufresne M. Hammond-Kosack K. E. Richard-Forget F. Barreau C. (2012). The velvet gene, FgVe1, affects fungal development and positively regulates trichothecene biosynthesis and pathogenicity in *Fusarium graminearum*. Mol. Plant Pathol. 13, 363–374. doi: 10.1111/j.1364-3703.2011.00755.x, 22013911 PMC6638759

[ref96] Mészáros B. Erdos G. Dosztányi Z. (2018). IUPred2A: context-dependent prediction of protein disorder as a function of redox state and protein binding. Nucleic Acids Res. 46, W329–W337. doi: 10.1093/nar/gky384, 29860432 PMC6030935

[ref97] Moon H. Lee M. K. Bok I. Bok J. W. Keller N. P. Yu J. H. (2023). Unraveling the gene regulatory networks of the global regulators VeA and LaeA in *Aspergillus nidulans*. Microbiol. Spectr. 11:e0016623. doi: 10.1128/spectrum.00166-23 36920196 PMC10101098

[ref98] Muraguchi H. Umezawa K. Niikura M. Yoshida M. Kozaki T. Ishii K. . (2015). Strand-specific RNA-seq analyses of fruiting body development in *Coprinopsis cinerea*. PLoS One 10:e0141586. doi: 10.1371/journal.pone.0141586, 26510163 PMC4624876

[ref99] Myung K. Li S. Butchko R. A. E. Busman M. Proctor R. H. Abbas H. K. . (2009). FvVE1 regulates biosynthesis of the mycotoxins fumonisins and fusarins in *Fusarium verticillioides*. J. Agric. Food Chem. 57, 5089–5094. doi: 10.1021/jf900783u, 19382792 PMC2692565

[ref100] Ni M. Yu J. H. (2007). A novel regulator couples sporogenesis and trehalose biogenesis in *Aspergillus nidulans*. PLoS One 2:e970. doi: 10.1371/journal.pone.0000970, 17912349 PMC1978537

[ref101] Niu J. Arentshorst M. Nair P. D. Dai Z. Baker S. E. Frisvad J. C. . (2015). Identification of a classical mutant in the industrial host *Aspergillus niger* by systems genetics: LaeA is required for citric acid production and regulates the formation of some secondary metabolites. G3 (Bethesda) 6, 193–204. doi: 10.1534/g3.115.024067, 26566947 PMC4704718

[ref102] O’Callaghan J. Coghlan A. Abbas A. García-Estrada C. Martín J. F. (2013). Functional characterization of the polyketide synthase gene required for ochratoxin a biosynthesis in *Penicillium verrucosum*. Intern. J. Food Microbiol. 161, 172–181. doi: 10.1016/j.ijfoodmicro.2012.12.014, 23334095

[ref103] Oakley C. E. Ahuja M. Sun W. W. Entwistle R. Akashi T. Yaegashi J. . (2017). Discovery of McrA, a master regulator of *Aspergillus* secondary metabolism. Mol. Microbiol. 103, 347–365. doi: 10.1111/mmi.13562, 27775185 PMC5218965

[ref104] Oda K. Kobayashi A. Ohashi S. Sano M. (2011). *Aspergillus oryzae* laeA regulates kojic acid synthesis genes. Biosci. Biotechnol. Biochem. 75, 1832–1834. doi: 10.1271/bbb.110235, 21897021

[ref105] Odoni D. I. Vazquez-Vilar M. Van Gaal M. P. Schonewille T. Martíns dos Santos V. A. P. Tamayo-Ramos J. A. . (2019). *Aspergillus niger* citrate exporter revealed by comparison of two alternative citrate producing conditions. FEMS Microbiol. Letters 366:fnz071. doi: 10.1093/femsle/fnz071, 31062025 PMC6502548

[ref106] Palmer J. M. Theisen J. M. Duran R. M. Grayburn W. S. Calvo A. M. Keller N. P. (2013). Secondary metabolism and development is mediated by LlmF control of VeA subcellular localization in *Aspergillus nidulans*. PLoS Genet. 9:e1003193. doi: 10.1371/journal.pgen.1003193, 23341778 PMC3547832

[ref107] Paoletti M. Seymour F. Alcocer M. Kaur N. Calvo A. M. Archer D. B. . (2007). Mating type and the genetic basis of self-fertility in the model fungus *Aspergillus nidulans*. Curr. Biol. 17, 1384–1389. doi: 10.1016/j.cub.2007.07.012, 17669651

[ref108] Patananan A. N. Palmer J. M. Garvey G. S. Keller N. P. Clarke S. G. J. (2013). A novel automethylation reaction in the *Aspergillus nidulans* LaeA protein generates S-methylmethionine. Biol. Chem. 17, 14032–14045. doi: 10.1074/jbc.M113.465765, 23532849 PMC3656261

[ref109] Perrin R. M. Fedorova N. D. Bok J. W. Cramer R. A. Wortman J. R. Kim H. S. . (2007). Transcriptional regulation of chemical diversity in *Aspergillus fumigatus* by LaeA. PLoS Pathog. 3:e50. doi: 10.1371/journal.ppat.0030050, 17432932 PMC1851976

[ref110] Price M. S. Yu J. Nierman W. C. Kim H. S. Pritchard B. Jacobus C. A. . (2006). The aflatoxin pathway regulator AflR induces gene transcription inside and outside of the aflatoxin biosynthetic cluster. FEMS Microbiol. Lett. 255, 275–279. doi: 10.1111/j.1574-6968.2005.00084.x, 16448506

[ref111] Reino J. L. Guerrero R. F. Hernández-Galán R. Collado I. G. (2008). Secondary metabolites from species of the biocontrol agent *Trichoderma*. Phytochem. Rev. 7, 89–123. doi: 10.1016/j.fbr.2016.05.001

[ref112] Reyes-Domínguez Y. Bok J. W. Berger H. Shwab E. S. Basheer A. Gallmetzer A. . (2010). Heterochromatic marks are associated with the repression of secondary metabolism clusters in *Aspergillus nidulans*. Mol. Microbiol. 76, 1376–1386. doi: 10.1111/j.1365-2958.2010.07051.x, 20132440 PMC2904488

[ref113] Sarikaya-Bayram O. Bayram O. Feussner K. Kim J. H. Kim H. S. Kaever A. . (2014). Membrane-bound methyltransferase complex VapA-VipC-VapB guides epigenetic control of fungal development. Dev. Cell 29, 406–420. doi: 10.1016/j.devcel.2014.03.020, 24871947

[ref114] Sarikaya-Bayram O. Dettmann A. Karahoda B. Moloney N. M. Ormsby T. McGowan J. . (2019). Control of development, secondary metabolism and light-dependent carotenoid biosynthesis by the velvet complex of *Neurospora crassa*. Genetics 212, 691–710. doi: 10.1534/genetics.119.302277, 31068340 PMC6614901

[ref115] Schalamun M. Beier S. Hinterdobler W. Wanko N. Schinnerl J. Brecker L. . (2024). MAPkinases regulate secondary metabolism, sexual development and light dependent cellulase regulation in *Trichoderma reesei*. Sci. Rep. 13:1912. doi: 10.1038/s41598-023-28938-w, 36732590 PMC9894936

[ref116] Schumacher J. Simon A. Cohrs K. C. Traeger S. Porquier A. Dalmais B. . (2015). The VELVETc in the gray mold fungus *Botrytis cinerea*: impact of BcLAE1 on differentiation, secondary metabolism, and virulence. Mol. Plant-Microbe Interact. 28, 659–674. doi: 10.1094/MPMI-12-14-0411-R, 25625818

[ref117] Seiboth B. Aghcheh R. Phatale P. A. Linke R. Hartl L. Sauer D. G. . (2012). The putative protein methyltransferase LAE1 controls cellulase gene expression in *Trichoderma reesei*. Mol. Microbiol. 84, 1150–1164. doi: 10.1111/j.1365-2958.2012.08083.x, 22554051 PMC3370264

[ref118] Shi J. C. Shi W. L. Zhou Y. R. Chen X. L. Zhang Y. Z. Zhang X. . (2020). The putative methyltransferase TlLAE1 is involved in the regulation of peptaibols production in the biocontrol fungus *Trichoderma longibrachiatum* SMF2. Front. Microbiol. 11:1267. doi: 10.3389/fmicb.2020.01267, 32612590 PMC7307461

[ref119] Steiger M. G. Rassingera A. Mattanovicha D. Sauera M. (2019). Engineering of the citrate exporter protein enables high citric acid production in *Aspergillus niger*. Metabol. Eng. 52, 224–231. doi: 10.1016/j.ymben.2018.12.004 30553933

[ref120] Stinnett S. M. Espeso E. A. Cobeño L. Araújo-Bazán L. Calvo A. M. (2007). *Aspergillus nidulans* VeA subcellular localization is dependent on the importin alpha carrier and on light. Mol. Microbiol. 63, 242–255. doi: 10.1111/j.1365-2958.2006.05506.x, 17163983

[ref121] Strauss J. Reyes-Domínguez Y. (2011). Regulation of secondary metabolism by chromatin structure and epigenetic codes. Fungal Genet. Biol. 48, 62–69. doi: 10.1016/j.fgb.2010.07.009, 20659575 PMC3935439

[ref122] Strohdiek A. Köhler A. M. Harting R. Stupperich H. Gerke J. Bastakis E. . (2025). The *Aspergillus nidulans* velvet domain containing transcription factor VeA is shuttled from cytoplasm into nucleus during vegetative growth and stays there for sexual development, but has to return into cytoplasm for asexual development. PLoS Genet. 21:e1011687. doi: 10.1371/journal.pgen.1011687, 40523015 PMC12169562

[ref123] Sugui J. A. Pardo J. Chang Y. C. Müllbacher A. Zarember K. A. Galvez E. M. . (2007). Role of LaeA in the regulation of alb1, gliP, conidial morphology, and virulence in *Aspergillus fumigatus*. Eukaryot. Cell 6, 1552–1561. doi: 10.1128/EC.00140-07, 17630330 PMC2043373

[ref124] Takao K. Akagi Y. Tsuge T. Harimoto Y. Yamamoto M. Kodama M. (2016). The global regulator LaeA controls biosynthesis of host-specific toxins, pathogenicity and development of *Alternaria alternata* pathotypes. J. Gen. Plant Pathol. 82, 121–131. doi: 10.1007/s10327-016-0656-9

[ref125] Tang W. Liu H. W. Zhao W. M. Wei D. Z. Zhong J. J. (2006). Ganoderic acid T from *Ganoderma lucidum* mycelia induces mitochondria mediated apoptosis in lung cancer cells. Life Sci. 80, 205–211. doi: 10.1016/j.lfs.2006.09.001, 17007887

[ref126] Teijeira F. Ullán R. V. Guerra S. M. García-Estrada C. Vaca I. Martín J. F. (2009). The transporter CefM involved in translocation of biosynthetic intermediates is essential for cephalosporin production. Biochem. J. 418, 113–124. doi: 10.1042/BJ20081180, 18840096

[ref127] Terfehr D. Dahlmann T. A. Kück U. (2017). Transcriptome analysis of the two unrelated fungal beta lactam producers *Acremonium chrysogenum* and *Penicillium chrysogenum*: velvet-regulated genes are major targets during conventional strain improvement programs. BMC Genomics 18:272. doi: 10.1186/s12864-017-3663-0, 28359302 PMC5374653

[ref128] Tsuge T. Harimoto Y. Akimitsu K. Ohtani K. Kodama M. Akagi Y. . (2013). Host-selective toxins produced by the plant pathogenic fungus *Alternaria alternata*. FEMS Microbiol. Rev. 37, 44–66. doi: 10.1111/j.1574-6976.2012.00350.x, 22846083

[ref129] Tsunematsu Y. Takanishi J. Asai S. Masuya T. Nakazawa T. Watanabe K. (2019). Genomic mushroom hunting decrypts coprinoferrin, a siderophore secondary metabolite vital to fungal cell development. Org. Lett. 21, 7582–7586. doi: 10.1021/acs.orglett.9b02861, 31496254

[ref130] Ullán R. V. Liu G. Casqueiro J. Gutiérrez S. Bañuelos O. Martín J. F. (2002). The cefT gene of *Acremonium chrysogenum* C10 encodes a putative multidrug efflux pump protein that significantly increases cephalosporin C production. Mol. Genet. Genomics 267, 673–683. doi: 10.1007/s00438-002-0702-5, 12172807

[ref131] Ullan R. V. Teijeira F. Guerra S. M. Vaca I. Martín J. F. (2010). Characterization of a novel peroxisome membrane protein essential for conversion of isopenicillin N into cephalosporin C. Biochem. J. 432, 227–236. doi: 10.1042/BJ20100827, 20819073

[ref132] Van Drogen F. Stucke V. M. Jorritsma G. Peter M. (2001). MAP kinase dynamics in response to pheromones in budding yeast. Nature Cell Biol. 3, 1051–1059. doi: 10.1038/ncb1201-1051, 11781566

[ref133] Veerappan C. S. Avramova Z. Moriyama E. N. (2008). Evolution of SET-domain protein families in the unicellular and multicellular *Ascomycota* fungi. BMC Evol. Biol. 8:190. doi: 10.1186/1471-2148-8-190, 18593478 PMC2474616

[ref134] Veiga T. Nijland J. G. Driessen A. J. Bovenberg R. A. Touw H. Van den Berg M. A. . (2012). Impact of velvet complex on transcriptome and penicillin G production in glucose limited chemostat cultures of a beta-lactam high-producing *Penicillium chrysogenum* strain. OMICS 16, 320–333. doi: 10.1089/omi.2011.0153, 22439693 PMC3369278

[ref135] Wang H. Guo Y. Luo Z. Gao L. Li R. Zhang Y. . (2022). Recent advances in *Alternaria* phytotoxins: A Review of their occurrence, structure, bioactivity, and biosynthesis. J. Fungi (Basel) 8:168. doi: 10.3390/jof8020168, 35205922 PMC8878860

[ref136] Wang Y. Wang L. Liu F. Wang Q. Selvaraj J. N. Xing F. . (2016). Ochratoxin A producing fungi, biosynthetic pathway and regulatory mechanisms. Toxins (Basel) 8:83. doi: 10.3390/toxins8030083, 27007394 PMC4810228

[ref137] Wang G. Zhang H. Wang Y. Liu F. Li E. Ma J. . (2019). Requirement of LaeA, VeA, and VelB on asexual development, ochratoxin a biosynthesis, and fungal virulence in *Aspergillus ochraceus*. Front. Microbiol. 10:2759. doi: 10.3389/fmicb.2019.02759, 31849898 PMC6892948

[ref138] Wiemann P. Brown D. W. Kleigrewe K. Bok J. W. Keller N. P. Humpf H. U. . (2010). FfVel1 and FfLae1, components of a velvet-like complex in fusarium fujikuroi, affect differentiation, secondary metabolism and virulence. Mol. Microbiol. 77, 972–994. doi: 10.1111/j.1365-2958.2010.07263.x, 20572938 PMC2989987

[ref139] Wong Sak Hoi J. Dumas B. (2010). Ste12 and Ste12-like proteins, fungal transcription factors regulating development and pathogenicity. Eukaryot. Cell 9, 480–485. doi: 10.1128/EC.00333-09, 20139240 PMC2863410

[ref140] Wu D. Oide S. Zhang N. Choi M. Y. Turgeon B. G. (2012). ChLae1 and ChVel1 regulate T-toxin production, virulence, oxidative stress response, and development of the maize pathogen *Cochliobolus heterostrophus*. PLoS Pathog. 8:e1002542. doi: 10.1371/journal.ppat.1002542, 22383877 PMC3285592

[ref141] Xing D. Deng C. Hu C. H. (2010). Molecular cloning and characterization of the regulator LaeA in *Penicillium citrinum*. Biotechnol. Lett. 32, 1733–1737. doi: 10.1007/s10529-010-0375-9, 20697928

[ref142] Yang Q. Chen Y. Ma Z. (2013). Involvement of BcVeA and BcVelB in regulating conidiation, pigmentation and virulence in *Botrytis cinerea*. Fungal Genet. Biol. 50, 63–71. doi: 10.1016/j.fgb.2012.10.003, 23147398

[ref143] Yu J. Chang P. K. Bhatnagar D. Cleveland T. E. (2000). Genes encoding cytochrome P450 and monooxygenase enzymes define one end of the aflatoxin pathway. Gene cluster in *Aspergillus parasiticus*. Appl. Microbiol. Biotechnol. 53, 583–590. doi: 10.1007/s002530051660, 10855719

[ref144] Zhang J. Chen H. Sumarah M. W. Gao Q. Wang D. Zhang Y. (2018). The veA gene acts as a positive regulator of conidia production, ochratoxin a biosynthesis, and oxidative stress tolerance in *Aspergillus niger*. J. Agric. Food Chem. 66, 13199–13208. doi: 10.1021/acs.jafc.8b04523, 30456955

[ref145] Zhang X. Hu Y. Liu G. Liu M. Li Z. Zhao J. . (2022). The complex Tup1-Cyc8 bridges transcription factor ClrB and putative histone methyltransferase LaeA to activate the expression of cellulolytic genes. Mol. Microbiol. 117, 1002–1022. doi: 10.1111/mmi.14885, 35072962

[ref146] Zhang X. Lia M. Zhu Y. Yang L. Qua J. Wang L. . (2020). *Penicillium oxalicum* putative methyltransferase Mtr23B has similarities and differences with LaeA in regulating conidium development and glycoside hydrolase gene expression. Fungal Gen. Biol. 143:103445. doi: 10.1016/j.fgb.2020.103445, 32822857

[ref147] Zhang M.-Y. Miyake T. (2009). Development and media regulate alternative splicing of a methyltransferase pre-mRNA in *Monascus pilosus*. J. Agric. Food Chem. 57, 4162–4167. doi: 10.1021/jf9004109, 19368389

[ref148] Zhang M. Yang Y. Li L. Liu S. Xue X. Gao Q. . (2022). LaeA regulates morphological development and ochratoxin a biosynthesis in *Aspergillus niger*. Mycotoxin Res. 38, 221–229. doi: 10.1007/s12550-022-00463-1, 35879501

[ref149] Zhao Z. Gu S. Liu D. Liu D. Chen B. Li J. . (2023). The putative methyltransferase LaeA regulates mycelium growth and cellulase production in *Myceliophthora thermophila*. Biotechnol. Biofuels Bioprod. 16:58. doi: 10.1186/s13068-023-02313-3, 37013645 PMC10071736

