# Supplementary material for: The global regulator LaeA controls expression of genes involved in diverse primary and secondary metabolites biosynthesis and their secretion in fungi
Source: Front Microbiol. 2026 Mar 9;17:1764309. doi: 10.3389/fmicb.2026.1764309 (PMC13006574; doi:10.3389/fmicb.2026.1764309)
Supplement: Supplementary file 1 [file Table_1.docx]

Supplementary Table 1				
**Fungi**	**Protein**	**Lenght (aa)**	**Position of the S-adenosyl-L-methionine-dependent methyltransferase Domain**	**Position of the S-adenosylmethionine binding sites**
Aspergillus nidulans	>XP_658411.2 protein laeA [Aspergillus nidulans FGSC A4]	374	84-317	144-150; 168-169; 187-189; 206
Aspergillus fumigatus	>AAR01218.1 LaeA [Aspergillus fumigatus]	349	64-291	118-124; 142-143; 161-163
Aspergillus flavus	>AAX68412.1 methyltransferase LaeA [Aspergillus flavus]	369	71-311	139-145; 163-164; 182-184; 201
Aspergillus parasiticus	>KAK6838914.1 Secondary metabolism regulator laeA [Aspergillus parasiticus]	370	71-311	139-145; 163-164; 182-184; 201
Aspergillus carbonarius	>OOF95411.1 hypothetical protein ASPCADRAFT_5941 [Aspergillus carbonarius ITEM 5010]	357	75-354	125-131; 149-150; 168-170; 187
Aspergillus terreus	>GFF14602.1 regulator of secondary metabolism LaeA [Aspergillus terreus]	354	63-296	123-129; 147-148; 166-168; 185
Aspergillus niger	>GAQ36052.1 methyltransferase [Aspergillus niger]	342	97-281	109-115; 133-134; 152-154
Aspergillus oryzae	>BAF74528.1 putative methyltransferase LAEA [Aspergillus oryzae]	369	71-311	139-145; 163-164; 182-184; 201
Aspergillus luchuensis	>XP_041540493.1 class I SAM-dependent methyltransferase [Aspergillus luchuensis]	344	61-339	111-117; 135-136; 154-156; 173
Aspergillus fumisynnematus	>AKP07636.1 LAEA [Aspergillus fumisynnematus]	373	83-315	142-148; 166-167; 185-187
Penicillium chrysogenum	>ACD50375.1 secondary metabolism regulator LaeA [Penicillium chrysogenum]	427	137-352	195-201;219-220; 238-240
Penicillium citrinum	>KAK5797658.1 hypothetical protein VI817_003949 [Penicillium citrinum]	273	28-270	42-48; 66-67; 85-87
Penicillium oxalicum	>EPS25650.1 hypothetical protein PDE_00584 [Penicillium oxalicum 114-2]	348	60-289	117-123; 141-142; 160-162
Acremonium chrysogenum	>KFH43747.1 Phosphomethylethanolamine N-methyltransferase-like protein [Hapsidospora chrysogenum ATCC 11550] (Acremonium chrysogenum)	350	88-348	131-137; 155-156; 173-175; 191
Fusarium fujikuroi	>VTT57528.1 unnamed protein product [Fusarium fujikuroi]	385	104-383	158-164; 182-183; 200-202; 218
Fusarium verticillioides	>RBQ91153.1 hypothetical protein FVER53263_12577 [Fusarium verticillioides]	346	50-344	103-109; 127-128; 145-147; 163
Fusarium oxysporum	>XP_059465472.1 S-adenosyl-L-methionine-dependent methyltransferase [Fusarium oxysporum Fo47]	327	41-316	Not defined
Fusarium graminearum	>WXC52520.1 hypothetical protein QX201_012177 [Fusarium graminearum]	334	50-331	103-109; 127-128; 145-147; 163
Trichoderma longibrachiatum	18648]	355	78-354	127-133; 151-152; 169-171; 188
Trichoderma reesei	>AFX86442.1 LAE1 [Trichoderma reesei]	365	35-269	107-113; 132-133; 150-152; 170
Alternaria alternata	>OWY52043.1 methyltransferase [Alternaria alternata]	310	25-308	78-84; 102; 121-123; 140
Cochliobolus heterostrophus	>KAJ6276793.1 methyltransferase [Bipolaris maydis] (Cochliobolus heterostrophus)	352	63-293	121-127; 145-146; 163-165; 182
Monascus pilosus	>sp|A2SUH3.1|LAEA_MONPI RecName: Full=Secondary metabolism regulator laeA; AltName: Full=Methyltransferase laeA; [Monascus pilosus]	367	80-309	136-142; 160-161; 179-181; 198
Monascus ruber	>AIY63188.1 LaeA [Monascus ruber]	368	81-310	137-143; 161-162; 180-182; 199
Coprinopsis cinerea	>XP_001829319.1 hypothetical protein CC1G_00498 [Coprinopsis cinerea okayama7#130]	402	91-322	155-161; 179-180; 198-200; 217
Myceliophthora thermophila	>KAL2184374.1 S-adenosyl-L-methionine-dependent methyltransferase [Thermothelomyces heterothallicus	314	34-253	91-97; 116-117; 134-136; 154
Cladosporium fulvum	>XP_047760139.1 Methyltransferase pytC [Fulvia fulva] (Cladosporium fulvum)	344	65-341	114-120; 138-139; 156-158; 175
Botrytis cinerea	>KAK6612086.1 methyltransferase domain-containing protein [Botrytis cinerea]	330	44-308	105-111; 133-134; 151-153; 171
